# NuMA regulates mitotic spindle assembly, structural dynamics and function via phase separation

**DOI:** 10.1038/s41467-021-27528-6

**Published:** 2021-12-09

**Authors:** Mengjie Sun, Mingkang Jia, He Ren, Biying Yang, Wangfei Chi, Guangwei Xin, Qing Jiang, Chuanmao Zhang

**Affiliations:** Key Laboratory of Cell Proliferation and Differentiation of the Ministry of Education, College of Life Sciences, Peking University, 100871 Beijing, China

**Keywords:** Chromosome segregation, Mitotic spindle, Microtubules, Mitotic spindle

## Abstract

A functional mitotic spindle is essential for accurate chromosome congression and segregation during cell proliferation; however, the underlying mechanisms of its assembly remain unclear. Here we show that NuMA regulates this assembly process via phase separation regulated by Aurora A. NuMA undergoes liquid-liquid phase separation during mitotic entry and KifC1 facilitates NuMA condensates concentrating on spindle poles. Phase separation of NuMA is mediated by its C-terminus, whereas its dynein-dynactin binding motif also facilitates this process. Phase-separated NuMA droplets concentrate tubulins, bind microtubules, and enrich crucial regulators, including Kif2A, at the spindle poles, which then depolymerizes spindle microtubules and promotes poleward spindle microtubule flux for spindle assembly and structural dynamics. In this work, we show that NuMA orchestrates mitotic spindle assembly, structural dynamics and function via liquid-liquid phase separation regulated by Aurora A phosphorylation.

## Abstract (web summary)

Mitotic spindle assembly is required for proper cell division, but many underlying mechanisms remain unclear. Here, the authors show that NuMa undergoes liquid-liquid phase separation, condensing on spindle poles during mitotic entry and enriching critical components to promote spindle assembly.

## Introduction

A well-assembled mitotic spindle is a dynamic macromolecular structure in which microtubules, microtubule-associated, and motor proteins tightly cooperate for its assembly, structural dynamics, and function. The mitotic spindle directly regulates accurate chromosome congression and segregation through correct connection with kinetochores assembled on both sites of each chromosome in mitosis and failure to do so may result in chromosome instability and abnormal cell division, leading to cell death or malignant cell proliferation^1–4^. The mitotic spindle assembly and structural dynamics are regulated by a variety of complicated mechanisms, among which the poleward spindle microtubule flux has been proposed to control the dynamic spindle length and structure maintenance via continuously adding tubulin subunits at the plus end of the spindle microtubule and flowing them to the minus end where they are removed^3,5–9^. Kinesin-13 family members are responsible for depolymerization of microtubules and provide important ways to regulate the spindle assembly and structural dynamics. Among the Kinesin-13 family members, Kif2A is concentrated mainly at the spindle poles and regulates the poleward spindle microtubule flux through depolymerizing the microtubules at their minus end during chromosome congression and segregation^8–11^. Nevertheless, although plenty of efforts have been paid, the underlying mechanisms for the mitotic spindle assembly, structural dynamics, and accurate function are still poorly understood.

NuMA (Nuclear Mitotic Apparatus) is a large spindle microtubule-associated protein that plays a dual role in organizing the mitotic spindle poles and controlling the spindle orientation in mitosis. It concentrates at the mitotic spindle poles controversially dependent on dynein proteins^12,13^, and depletion of NuMA or dynein may result in focus defects of the mitotic spindle poles^13,14^. In addition to its spindle pole localization and function in organization of the spindle poles, NuMA is also recruited to cell cortex in complex with dynein–dynactin by LGN^15^, where it regulates spindle orientation through forming dynein–dynactin–NuMA clusters at the mitotic cell cortex^16^. NuMA is phosphorylated by the mitotic kinases Aurora A and cyclin-dependent kinase-1 during mitosis^17–19^, through which its balanced dynamic localization and function on the spindle pole and the cell cortex are tightly regulated. NuMA molecule is roughly divided into a globular head, a tail domain, and a discontinuous coiled-coil middle domain with 1500 amino acid residues^20,21^. More likely due to too large in molecular weight and too complicated in molecular features, the underlying mechanisms for functions of NuMA remain largely mysterious so far.

It has been found recently that numerous cellular compartments are formed through liquid–liquid phase separation (LLPS) without surrounding membranes and play important roles in cellular activities^22^. When a protein concentration is saturated, LLPS may occur spontaneously to separate into two phases, a dilute phase and a dense phase^23^. The dense phase, called droplet or condensate, usually displays liquid-like properties. In some cases, the droplet may undergo a liquid-to-solid phase transition^24^. Germline P granule was first reported possessing liquid-like properties and is formed through phase separation^25^. In the past few years, an increasing number of proteins have been reported to display phase separation ability in vitro and in vivo, and through concentrating other passenger proteins that cannot phase-separate on their own, these protein droplets regulate a variety of cellular activities^26,27^. The concept of emergence of the membrane-less organelles offers new insights into the mechanisms of the mitotic spindle assembly and structural dynamics. BugZ, a spindle matrix protein, promotes mitotic spindle assembly through phase separation to concentrate tubulin proteins^28^. A liquid-like structure, called the liquid-like meiotic spindle domain, was also identified to promote acentrosomal spindle assembly in mammalian oocytes^29^. A more recent work also found that the microtubule-associated protein TPX2 promotes microtubule nucleation and elongation through phase separation^30^.

In this work, we unexpectedly find that the spindle pole-concentrated NuMA regulates mitotic spindle assembly, structural dynamics, and function through phase separation in a regulated way. We demonstrate that C-terminus of NuMA is responsible for its phase separation and that Aurora A phosphorylation of NuMA fluidizes NuMA for enhancement of its dynamics at the mitotic spindle poles and cell cortex via regulating its phase separation status. We discover that both phase separation of NuMA and KifC1 facilitate concentration of NuMA at the spindle poles and that this phase separation regulates the spindle assembly and the structural dynamics through sequestering Kif2A on the spindle poles. We also reveal that the binding of NuMA droplets to microtubules also facilitates sorting of acentrosomal microtubule asters into the spindle microtubule array for the mitotic spindle assembly and structural dynamics. Collectively, these data provide a molecular mechanism for spatiotemporal regulation of spindle assembly, structural dynamics, and function mediated by NuMA phase separation and this work may have more implications for understanding the mechanism of accurate chromosome distribution into two daughter cells regulated by NuMA during the cell division.

## Results

### NuMA regulates mitotic spindle assembly and structural dynamics

**Fig. 1:**
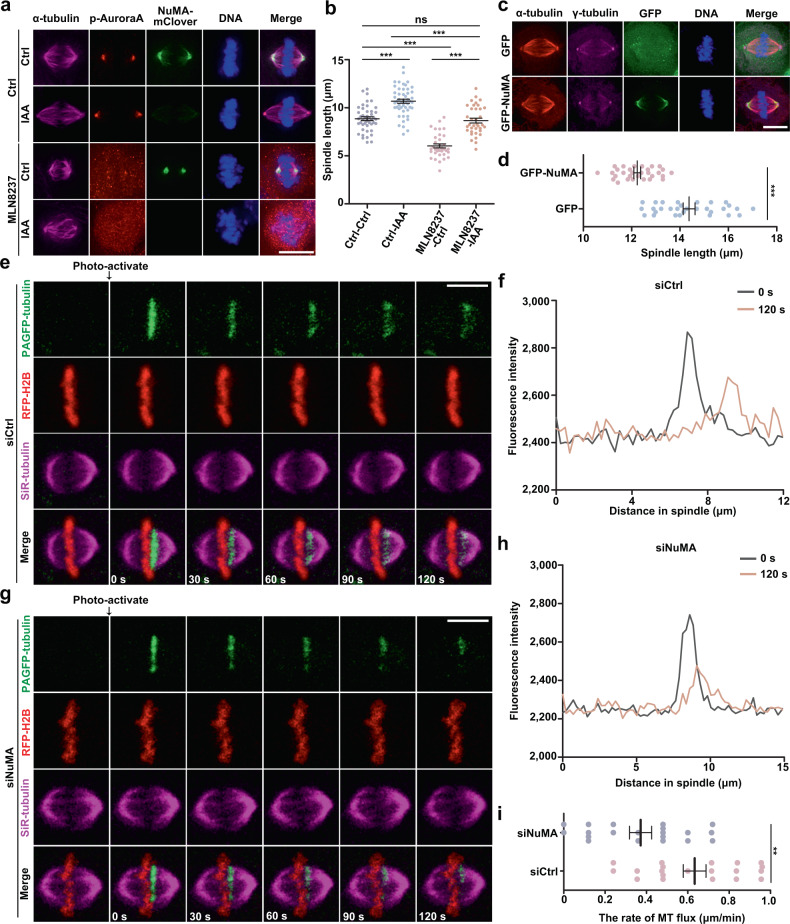
NuMA regulates mitotic spindle assembly and structural dynamics. **a** Immunofluorescence images of mitotic spindles upon NuMA depletion in the control and MLN8237-treated degron systems. NuMA-mACF cells were treated with 1 μg/ml Dox and 500 μM IAA for 24 h and then treated with 0.25 μM MLN8237 for 30 min, followed by immunostaining with anti-p-Aurora A and α-tubulin antibodies. DNA was stained with DAPI. **b** Quantification of spindle length in **a** (mean ± SEM, *n* = 46, 50, 35, 36 cells in one experiment, three independent experiments were repeated). Unpaired two-tailed *t* test: *P* < 0.0001 for Ctrl-Ctrl/Ctrl-IAA, Ctrl-Ctrl/MLN8237-Ctrl, Ctrl-IAA/MLN8237-IAA, MLN8237-Ctrl/MLN8237-IAA, and *P* = 0.5379 for Ctrl-Ctrl/MLN8237-IAA. **c** Immunofluorescence images of mitotic spindles in HeLa cells transiently expressing GFP or GFP-NuMA stained with anti-α-tubulin and γ-tubulin antibodies and DAPI. **d** Quantification of spindle length in **c** (mean ± SEM, *n* = 30 cells in 1 experiment, 3 independent experiments were repeated). Unpaired two-tailed *t* test: *P* < 0.0001 for GFP/GFP-NuMA. **e**, **g** Time-lapse images showing poleward spindle microtubule flux in HeLa cells stably expressing RFP-H2B and transiently expressing photoactivatable GFP-tagged α-tubulin (PAGFP-α-tubulin) with control or NuMA siRNA. Microtubules were probed with SiR-tubulin. The PAGFP signal in a rectangular region near the MT-plus ends was activated (time point 0, arrows) and tracked every 10 s. **f**, **h** Fluorescence intensity profiles at time points from 0 and 120 s in **e**, **g**, respectively. **i** The mean velocity of poleward spindle microtubule flux in control and NuMA siRNA HeLa cells. The mean velocity was defined by the ratio of the distance that the PAGFP signal travels to the time it takes (mean ± SEM, *n* = 20 cells in 1 experiment, 2 independent experiments were repeated). Unpaired two-tailed *t* test: *P* = 0.0016 for siCtrl/siNuMA. **P < 0.01, and ***P < 0.001, ns, not significant. Scale bars, 10 μm.

To investigate the molecular mechanisms underlying mitotic spindle assembly, structural dynamics, and function, we first depleted NuMA in HCT-116 cells by using the auxin-inducible degron (AID) system, which knocks in a specific vector, mAID-mClover-FLAG, that fuses to endogenous NuMA (NuMA-mACF) to promote efficient endogenous NuMA protein degradation under doxycycline (Dox) and 3-indoleacetic acid (IAA) induction^16^ and investigated the consequences of NuMA depletion. Interestingly, we observed that endogenous NuMA depletion resulted in significant longer metaphase spindle formation than the non-depletion control (Fig. 1a, b). Then we treated HeLa cells with 0.25 μM MLN8237 to inhibit the kinase activity of Aurora A without influencing activity of Aurora B. More strikingly, we found that Aurora A inhibition did not shorten the spindle length in NuMA-depleted cells (Fig. 1a, b and Supplementary Fig. 1a, b). In verifying this, we observed that overexpression of NuMA significantly reduced spindle length (Fig. 1c, d). Previous studies suggested that microtubule flux regulates spindle length in several systems^7,8,31^. Hence, we tested whether NuMA depletion affected microtubule flux. Through NuMA RNA interference (RNAi) knockdown in HeLa cells expressing photoactivatable green fluorescent protein (PA-GFP) tubulin and live-cell imaging, we observed that the microtubule flux rates of the mitotic spindle were significantly reduced in NuMA-depleted cells compared with that in control cells, leading to longer spindle formation (Fig. 1e–i, Supplementary Fig. 1c, and Supplementary Movies 1 and 2). In parallel, NuMA depletion also induced abnormalities of mitotic spindle assembly, chromosome congression, and segregation (Supplementary Fig. 1d–g). To rule out that chromosome congression defects cause elongation of the spindles, we also measured the spindle length in cells with normal chromosome congression and the half spindle length in cells with abnormal chromosome congression after NuMA depletion (Supplementary Fig. 1h, i). The results showed that spindle microtubules were still elongated in the both conditions, indicating that the growing spindle length was due to microtubule elongation rather than chromosome congression defects. These results suggested that NuMA involves in mitotic spindle assembly and dynamics through regulating the microtubule flux of the mitotic spindle.

Based on all of the results above, we conclude that NuMA regulates mitotic spindle assembly, structural dynamics, and spindle length by affecting poleward spindle microtubule flux under the regulation of Aurora A.

### NuMA phase-separates during mitosis

**Fig. 2:**
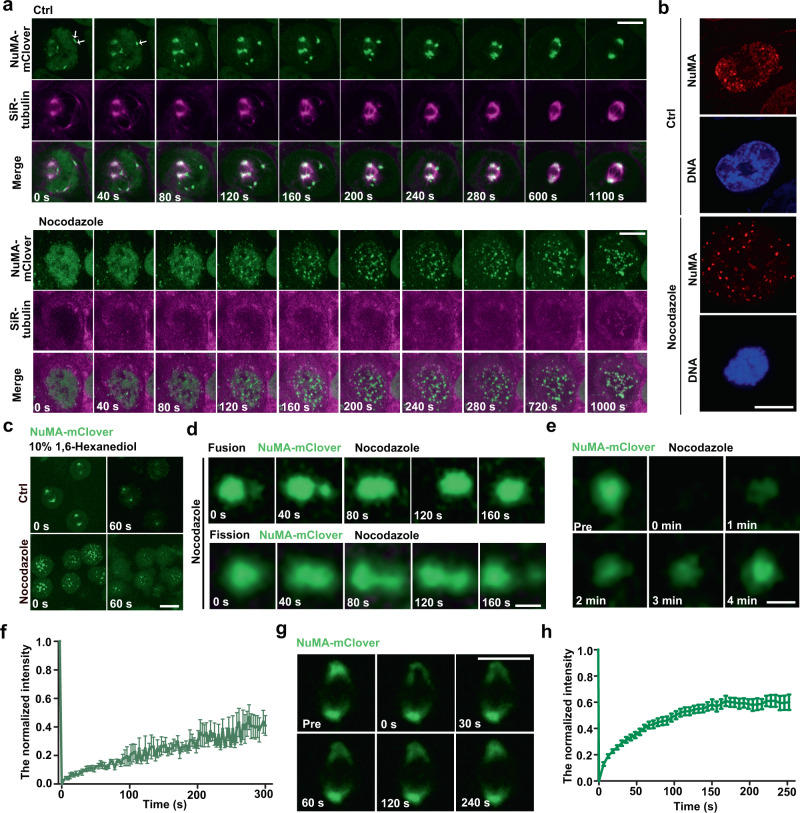
NuMA phase-separates during G2/M transition. **a** Time-lapse images showing endogenous NuMA tagged with mClover forms droplets spontaneously along with NEBD in both control and nocodazole-treated NuMA-mACF cells. Images were collected every 40 s. Microtubules were probed by SiR-tubulin. The arrows indicated the NuMA droplet fusion. **b** Immunofluorescence images showing the endogenous NuMA condensates in control or nocodazole-treated mitotic HeLa cells, followed by staining with anti-α-NuMA antibody (red) and DAPI (blue) for DNA. **c** Time-lapse images showing the dynamics of NuMA droplets in NuMA-mACF cells. Note that NuMA droplets were located to spindle poles in cells without nocodazole treatment and to the cytoplasm when the cells were treated with nocodazole and that the droplets disappeared in both nocodazole- and non-nocodazole-treated cells within 60 s in the presence of 10% hexanediol. **d** Time-lapse imaging showing fusion and fission of endogenous NuMA-mClover droplets in nocodazole-treated mitotic NuMA-mACF cells. Scale bar, 1 μm. **e**, **f** Time-lapse images (**e**) and quantification (**f**) of fluorescence recovery after photobleaching (FRAP) of endogenous NuMA-mClover droplets after photobleaching in nocodazole-treated NuMA-mACF cells (mean ± SEM, *n* = 6 droplets). Scale bar, 1 μm. **g**, **h** Time-lapse images (**g**) and quantification (**h**) of FRAP of endogenous NuMA-mClover on the spindle pole after photobleaching in NuMA-mACF cells (mean ± SEM, *n* = 20 cells). Scale bars, 10 μm unless specified otherwise.

Poleward spindle microtubule flux is mainly achieved through microtubule polymerization at its plus end connected with chromosome kinetochores and depolymerization at its minus end situated at the spindle poles^6,8,32,33^. To determine how NuMA affects the poleward spindle microtubule flux, we first investigated how NuMA accumulates at the spindle poles. It was reported that overexpressed exogenous NuMA formed numerous condensates along with nuclear envelope breakdown (NEBD)^34^, and these NuMA condensates promoted the initial step of spindle bipolarization in early mitosis by organizing microtubule asters regardless of the existence of centrosomes^35^. Our results showed that both endogenous NuMA tagged with mClover in HCT-116 cells and exogenous GFP-NuMA stably expressed in HeLa cells spontaneously assembled into many droplets along with NEBD in cells, and these droplets showed liquid-like behaviors by flowing, fusing with each other, and were finally transported to the spindle poles along with microtubules to form big droplets at the spindle poles (Fig. 2a, Supplementary Fig. 2a, and Supplementary Movie 3). To further investigate the physical property of the droplets, we depolymerized microtubules with 100 ng/ml nocodazole. In nocodazole-treated cells, NuMA formed droplets independent of both the NE and the microtubules; expectedly, they could not move to the spindle poles without microtubules (Fig. 2a, Supplementary Fig. 2a, and Supplementary Movie 4). Along with the emergence of NuMA condensates in nocodazole-treated cells, tubulin proteins were gradually loaded into the droplets. These results were also confirmed by immunofluorescence (IF) labeling in HeLa cells (Fig. 2b). To determine whether NuMA droplets are formed through LLPS, we investigated their sensitivity to 1,6-hexanediol, a compound known for destroying liquid-like droplet and inhibiting the droplet formation putatively through disrupting hydrophobic interactions. We found that 10% 1,6-hexanediol treatment of HCT-116 cells expressing endogenously tagged NuMA-mClover destroyed almost all NuMA droplets within 60 s in both control- and nocodazole-treated cells and no new NuMA droplets formed in the presence of 10% 1,6-hexanediol (Fig. 2c and Supplementary Movies 5 and 6). Furthermore, correlative confocal and electron microscopic images showed that the NuMA droplets were not closed by membranes (Supplementary Fig. 2b). Moreover, in nocodazole-treated HCT-116 and HeLa cells, both the endogenous and exogenous NuMA droplets underwent fusion and fission purely like liquid drops (Fig. 2d, Supplementary Fig. 2c, and Supplementary Movies 7 and 8). When fluorescence of these droplets was quenched through fluorescence recovery after photobleaching (FRAP), we observed that it was recovered quickly (Fig. 2e, f and Supplementary Fig. 2d, e), indicating that NuMA within the droplets exchanged with surrounding dispersed free NuMA frequently. NuMA at the spindle poles also displayed a similar recovery rate and degree to above-mentioned cytoplasmic droplets in the FRAP assay (Fig. 2g, h and Supplementary Fig. 2f, g), indicating that NuMA on spindle poles was assembled into viscous liquid droplets. Thus, we conclude that NuMA spontaneously forms viscous liquid droplets through phase separation during mitosis.

### C-terminus is required for NuMA phase separation

**Fig. 3:**
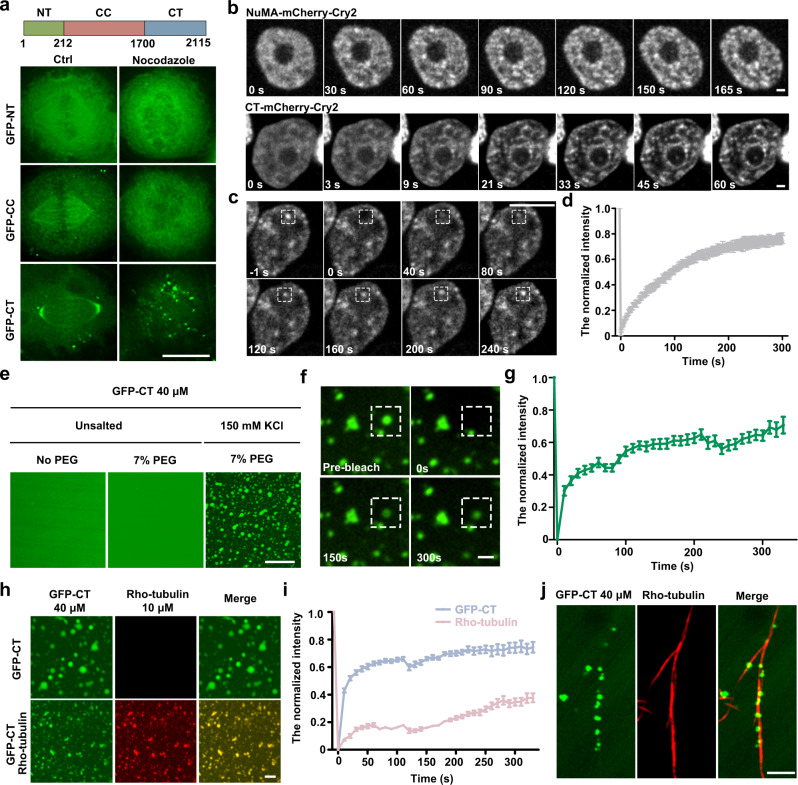
C-terminus of NuMA is required for mitotic phase separation. **a** Schematic diagram and immunofluorescence images of three domains of NuMA expressed in mitotic HeLa cells without (Ctrl) or with treatment of nocodazole. **b** Time-lapse images of HEK293T cells expressing full-length NuMA or CT mutant fused with mCherry-Cry2. The cells were subjected to laser excitation and images were captured every 3 s. Scale bar, 2 μm. **c**, **d** Time-lapse images (**c**) and quantification (**d**) of FRAP of CT droplets in OptoDroplet system (mean ± SEM, *n* = 12 droplets). **e** Confocal microscopic images of GFP-CT droplets in vitro. GFP-CT were incubated in phase separation assay buffer at the indicated conditions and visualized by confocal microscopy. **f**, **g** Time-lapse images (**f**) and quantification (**g**) of FRAP of GFP-CT droplets phase-separated in vitro (mean ± SEM, *n* = 13 droplets). Scale bar, 2 μm. **h** Confocal microscopic images of GFP-CT and Rho-tubulin droplets phase-separated in vitro. GFP-CT were incubated with Rho-tubulin in phase separation buffer at the indicated conditions, followed by visualization with confocal microscopy. Scale bar, 2 μm. **i** Quantifications of FRAP of GFP-CT/Rho-tubulin droplets phase-separated in vitro (mean ± SEM, *n* = 10 droplets per sample). **j** Microscopic images of GFP-CT droplets binding to taxol-stabilized microtubules in vitro. Taxol-stabilized microtubules were added to GFP-CT droplets in phase separation buffer and visualized with confocal microscopy. Scale bars, 10 μm unless specified otherwise.

We analyzed NuMA protein sequences using PONDR program to predict the disordered regions^36^. The N-terminus of NuMA (NT) was predicted to have ordered regions, while the C-terminal tail domain (CT) and the long coiled-coil middle domain (CC) were largely unstructured with disordered regions (Supplementary Fig. 3a). To determine which region of the NuMA molecule mediates its phase separation in vivo, we first generated GFP-tagged truncated mutants, including GFP-NT, GFP-CT, and GFP-CC, and expressed them in HeLa cells. The results showed only GFP-CT formed droplets during mitotic entry in both control- and nocodazole-treated mitotic HeLa cells, although the numbers of GFP-CT droplets were less than those of full-length NuMA droplets in cells expressing full-length NuMA (Fig. 3a and Supplementary Fig. 3b). In contrast, GFP-NT showed a smeared cytoplasmic distribution, and GFP-CC was bound to spindle microtubules but not at the spindle poles (Fig. 3a and Supplementary Fig. 3c, d). OptoDroplet system also showed that, under blue light induction, only the NuMA-mCherry-Cry2 and CT-mCherry-Cry2 proteins facilitated the rapid formation of micron-sized spherical droplets, whereas both the CC-mCherry-Cry2 and the negative control mCherry-Cry2 proteins did not form droplet structures under the same conditions (Fig. 3b and Supplementary Fig. 3e). Furthermore, under FRAP, the fluorescence intensity of the photoactivated CT-mCherry-Cry2 droplets recovered quickly (Fig. 3c, d).

Since the in vivo environment is complicated for NuMA in phase separation, we set to determine whether other proteins contribute to phase separation of NuMA. We purified GFP-CT proteins in vitro, and the analytical size-exclusion chromatography showed that purified GFP-CT proteins were mixed with monomers, oligomers, and high polymers due to the ability of its oligomerization^37^ (Supplementary Fig. 4a, b). Through an in vitro phase separation assay, we found that the purified GFP-CT efficiently assembled into micrometer-sized spherical droplets in the presence of a macromolecular crowding agent polyethylene glycol (PEG) and these in vitro GFP-CT droplets also rapidly recovered after photobleaching (Fig. 3e–g).

Based on these results, we conclude that NuMA can efficiently phase-separate alone, at least in vitro, and that its C-terminus is required for its phase separation, although other parts of the NuMA molecule also contribute to droplet formation.

### NuMA droplets sort microtubule asters to organize spindles

Since NuMA can bind microtubules in vivo, we incubated GFP-CT with rhodamine-tubulin in the above phase separation buffer to determine whether the phase-separated droplets concentrate tubulin proteins. While GFP-CT formed droplets alone in the buffer, the droplets efficiently concentrated Rho-tubulin when both GFP-CT and Rho-tubulin were coincubated (Fig. 3h). Furthermore, the fluorescence of GFP-CT rapidly recovered regardless of tubulin proteins, and the fluorescence of Rho-tubulin proteins in these droplets also recovered after bleaching (Fig. 3i). Moreover, when the phase-separated droplets were mixed with Taxol-stabilized microtubules in vitro, these droplets rapidly interacted with the microtubule fibers, although they obviously could not walk along the microtubule fibers (Fig. 3j), suggesting that the NuMA droplets need other motor proteins to poleward walk along the spindle microtubules.

Because NuMA droplets can concentrate tubulin in vitro, through a microtubule regrowth assay in NuMA-mACF cells, we set out to verify whether the NuMA droplets can nucleate microtubules. We found that the formation of acentrosomal microtubules in NuMA-depleted cells was as normal as that in control cells when the cells were released into fresh medium containing 15 ng/ml nocodazole from 500 ng/ml nocodazole (Supplementary Fig. 5a). However, when the NuMA-depleted cells were treated with 500 ng/ml nocodazole and then released into warmed fresh medium without nocodazole, we found that the acentrosomal microtubule asters failed to sort into the centrosomal microtubules for mitotic spindle assembly (Supplementary Fig. 5b, c).

Taken together, these results indicate that NuMA does not directly regulate acentrosomal microtubule nucleation and that its phase separation participates in concentrating tubulin proteins and sorting acentrosomal microtubules into the spindle microtubule array for mitotic spindle assembly.

### Aurora A phosphorylation fluidizes NuMA droplets

**Fig. 4:**
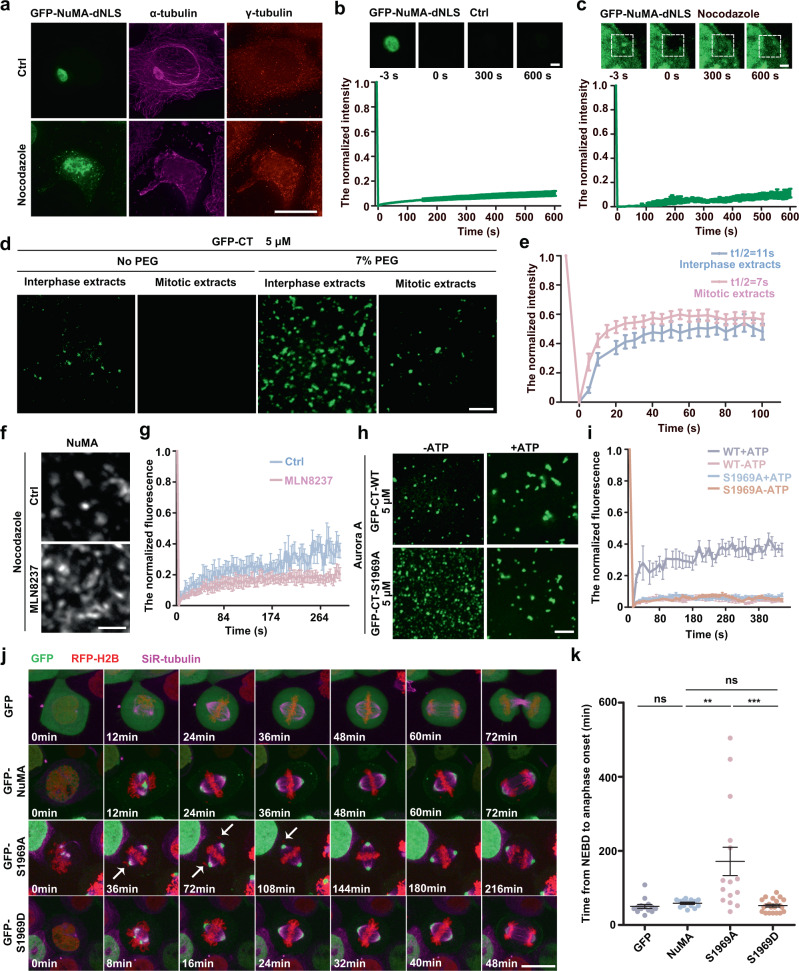
Phosphorylation by Aurora A regulates the phase separation status and function of NuMA in mitosis. **a** Immunofluorescence images showing the localization and features of GFP-NuMA-dNLS droplets in interphase HeLa cells transfected with GFP-NuMA-dNLS with or without nocodazole treatment. **b**, **c** Time-lapse images and quantification of FRAP of GFP-NuMA-dNLS droplets in mitotic HeLa cells without (**b**) or with nocodazole (**c**) treatment (mean ± SEM, *n* = 10 droplets per sample). Scale bars, 2 μm. **d** Phase separation of GFP-CT in interphase cytoplasmic extracts and mitotic cytoplasmic extracts under the indicated conditions, followed by visualization with confocal microscopy. **e** Quantification of FRAP of phase-separated GFP-CT droplets in interphase cytoplasmic extracts and mitotic extracts (mean ± SEM, *n* = 10 droplets per sample). **f** Immunofluorescence images of NuMA droplets in HeLa cells, showing the different morphologies. The cells were treated without (Ctrl) or with MLN8237, followed by treatment with nocodazole and staining with anti-NuMA antibody (gray). **g** Quantification of FRAP of endogenous NuMA-mClover droplets in the control or MLN8237-treated NuMA-mACF cells (mean ± SEM, *n* = 5 droplets for control and *n* = 11 droplets for MLN8237-treated cells). **h** Confocal microscopic images of GFP-CT or GFP-CT-S1969A droplets phase-separated in vitro in the mentioned conditions. GFP-CT or GFP-CT-S1969A proteins were incubated with kinase buffer containing Aurora A/ATP or Aurora A alone, followed by visualization with confocal microscopy. **i** Quantification of FRAP of GFP-CT or GFP-CT-S1969A droplets in (**h**) (mean ± SEM, *n* = 10, 8, 10, and 8 droplets for WT + ATP, WT-ATP, S1969A + ATP, and S1969A-ATP, respectively). **j** Time-lapse images of stable RFP-H2B-expressing HeLa cells transfected with GFP, GFP-NuMA, GFP-S1969A, and GFP-S1969D. Images were collected every 4 min. The white arrows indicate the aberrant congression of chromosomes. **k** The average time from NEBD to anaphase onset of the NuMA-expressing HeLa cells in **j** (mean ± SEM, *n* = 14, 15, 15, and 24 cells were measured for GFP, NuMA, S1969A, and S1969D, respectively). Unpaired two-tailed *t* test: *P* = 0.1723 for GFP/NuMA, *P* = 0.0083 for NuMA/S1969A, *P* = 0.2211 for NuMA/S1969D, *P* = 0.0006 for S1969A/S1969D. **P < 0.01, and ***P < 0.001, ns, not significant. Scale bars, 10 μm unless specified otherwise.

NuMA localized in nucleus in interphase and translocated to spindle poles during early mitosis. To compare the status of NuMA between interphase and mitosis so as to find the regulating mechanism, we generated and expressed GFP-tagged NLS (nuclear localization sequence)-deleting NuMA mutant (GFP-NuMA-dNLS) in HeLa cells. The results showed that GFP-NuMA-NLS was solely localized to the cytoplasm and formed large microtubule-containing spherical droplets; and when microtubules were depolymerized by nocodazole treatment, these large NuMA droplets were split into numerous smaller tubulin-containing droplets (Fig. 4a). However, under FRAP, these interphase NuMA droplets were less dynamic and more stable than mitotic NuMA droplets regardless of nocodazole treatment (Fig. 4b, c). To further prove that NuMA statuses in interphase and in mitosis were different, we performed experiments in vitro. The results showed that GFP-CT proteins formed less droplets when added to mitotic cell extracts than when added to interphase cell extracts; and under FRAP, the NuMA droplets recovered more rapidly in mitotic extracts than in interphase cytoplasmic extracts (Fig. 4d, e). These results showed that NuMA droplets would become solid in the absence of mitotic conditions. Therefore, we wondered what mechanisms maintained the liquid status of NuMA droplets in mitosis. Since posttranslational modifications (PTMs) regulate LLPS ability of proteins^38,39^, NuMA is highly phosphorylated at the C-terminus concomitant with NEBD^18–20,40^, and Aurora A directly phosphorylates C-terminus of NuMA^19,41^. We investigated the effects of PTMs on NuMA phase separation. Surprisingly, IF images and FRAP showed that inhibiting Aurora A with MLN8237 solidified NuMA droplets in nocodazole-treated mitotic cells (Fig. 4f, g). Since previous research showed that NuMA can be phosphorylated by Aurora A at S1969 and that Aurora A governs dynamic exchange between the cytoplasmic- and the spindle pole-localized pools of NuMA^19,41^, we investigated the effects of the phosphorylation by Aurora A on NuMA phase separation. We purified GFP-CT-S1969A proteins (Supplementary Fig. 4a), and using GFP-CT and GFP-CT-S1969A in the presence of Aurora A kinase and ATP, we performed a phase separation assay in vitro to investigate whether Aurora A directly regulates the phase separation of NuMA. Microscopic images showed that GFP-CT formed much less droplets in the presence of Aurora A and ATP (Fig. 4h). Meanwhile, FRAP experiments suggested that only the fluorescence intensity of GFP-CT droplets could recover under the phosphorylation by Aurora A, whereas GFP-CT-S1969A condensates were largely unrecoverable (Fig. 4i). To explore the function of Aurora A phosphorylation in vivo, we generated and individually overexpressed GFP, GFP-NuMA, GFP-NuMA-S1969D, and GFP-NuMA-S1969A in HeLa cells. We found that GFP-expressing cells did not change their cell cycle behavior, GFP-NuMA-S1969D-expressing cells showed a similar normal mitotic progression to GFP-NuMA-expressing cells, and both GFP-NuMA and GFP-NuMA-S1969D located not only to the spindle poles but also to the adjacent spindle microtubules and on the cell cortex in metaphase (Fig. 4j, k and Supplementary Movies 9 and 10). In contrast, GFP-NuMA-S1969A-expressing cells showed severely disturbed chromosome congression and significantly abnormal slow cell division, and GFP-NuMA-S1969A enriched to the spindle poles but not to the adjacent spindle microtubules and the cell cortex (Fig. 4j, k and Supplementary Movie 11). Collectively, we conclude that the phosphorylation of NuMA at S1969 by Aurora A reduces NuMA phase separation and fluidizes NuMA droplets during mitosis.

### KifC1 facilitates NuMA droplets to spindle poles

**Fig. 5:**
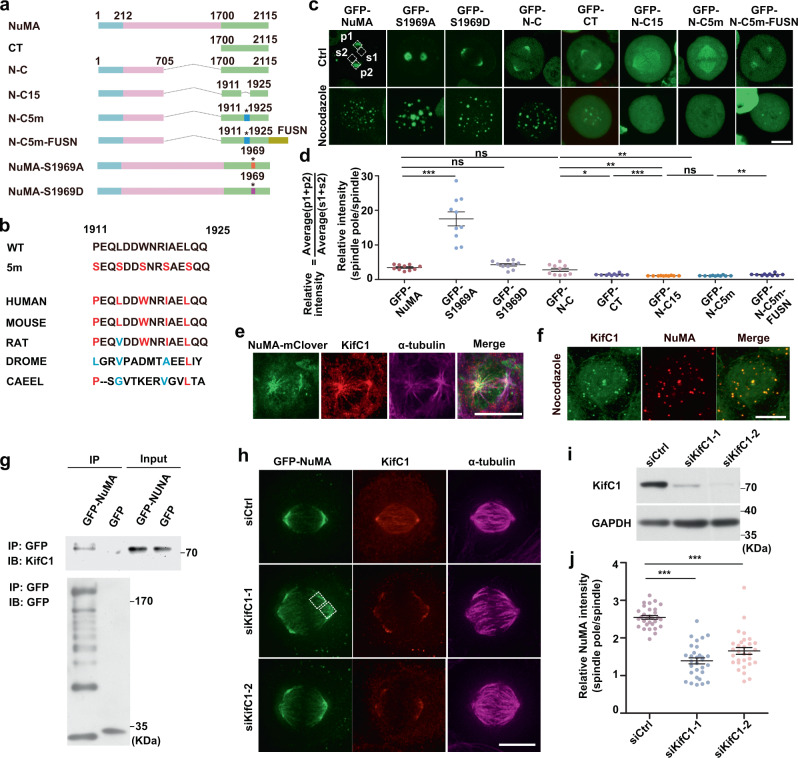
Phase separation and KifC1 facilitate NuMA to concentrate on spindle poles. **a** Schematic drawing of truncated and mutated NuMA constructs. Asterisks indicate the mutated sites. **b** Mutagenesis of five hydrophobic residues in N-C (black) to serine residues (red) in N-C5m. Multiple sequence alignment for the five hydrophobic amino acids was performed using Uniprot. Red amino acids were conserved and hydrophobic, and blue amino acids were not conserved but hydrophobic. **c** Live cell images of NuMA- or mutant-expressing HeLa cells. Four white dotted boxes of 3 × 3 μm denote areas to measure fluorescence intensity. P1, P2, S1, S2: the mean fluorescence intensity on drawing areas. **d** Schematic drawing and quantification of the relative fluorescence intensity of NuMA mutants on spindle poles in **c** (mean ± SEM, *n* = 10 cells per sample). Unpaired two-tailed *t* test: *P* < 0.0001 for NuMA/S1969A, *P* = 0.0824 for NuMA/S1969D, *P* = 0.2263 for NuMA/N-C, *P* = 0.0108 for N-C/CT, *P* = 0.0009 for CT/N-C15, *P* = 0.5996 for N-C15/N-C5m, *P* = 0.0020 for N-C/N-C15, *P* = 0.0022 for N-C/N-C5m, *P* = 0.0065 for N-C5m/N-C5m-FUSN. **e** Immunofluorescence images of the assembling spindle in NuMA-mACF cells stained with anti-KifC1 and α-tubulin antibodies. **f** Immunofluorescence images of mitotic HeLa cells treated with nocodazole and stained with anti-NuMA and KifC1 antibodies. **g** Immunoprecipitation assay showing that NuMA interacts with KifC1. HEK293T cells were transfected with GFP, GFP-NuMA, arrested in mitosis with nocodazole and processed for IP assay with GFP-Trap beads. **h** Immunofluorescence images of mitotic spindles in HeLa cells stably expressing GFP-NuMA with control or KifC1 siRNA, followed by immunostaining with anti-KifC1 and α-tubulin antibodies. The DNA was stained with DAPI. Two white dotted boxes of 3 × 3 μm denote areas to measure fluorescence intensity. **i** Immunoblotting for KifC1 and GAPDH expression in cell lysates with control and KifC1 siRNA knockdown. **j** Quantification of the relative NuMA fluorescence intensity on spindle poles in **h** (mean ± SEM, *n* = 30 cells per sample). The relative intensity was defined according to the method in **d**. Unpaired two-tailed *t* test: *P* < 0.0001 for siCtrl/siKifC1-1 and siCtrl/siKifC1-2. *P < 0.05, **P < 0.01, and ***P < 0.001, ns, not significant. Scale bars, 10 μm.

To determine the functional subdomain of NuMA molecule for its phase separation and the mechanism for transportation of NuMA droplets to the spindle poles, we first generated and expressed a series of truncated mutants in cells, followed by live cell imaging (Fig. 5a, b, Supplementary Fig. 6a). The truncation “N-C” (a combination of N-terminal dynein–dynactin-binding motif with CT) was found functioning as full-length NuMA^12,16^. Consistent with this, N-C formed droplets during early metaphase as effectively as full-length NuMA and more than CT (Fig. 5c, d), indicating other domains that cannot phase-separate alone could facilitate the ability of phase separation of C-terminus. Then we generated a series of deletion and point mutation mutants based on the N-C construct. We found that both N-C15, which lacks the aa1911–1925 motif, and N-C5m, which carries five hydrophobic mutations within aa1911–1925, were located evenly to the mitotic spindle but failed to localize to the mitotic spindle poles and abolished NuMA droplet formation (Fig. 5c, d). The important motif, which contains five hydrophobic amino acids, is conservative in mammals. Although the five residues in *drosophila* and *elegans* are not conserved to mammals, they are still hydrophobic, which might play the same roles. When the cells were treated with nocodazole, both N-C15 and N-C5m were evenly dispersed throughout the whole cellular area (Fig. 5c). Furthermore, when N-C5m was fused with FUSN (FUS N-terminus), its defects in droplet-formation ability and spindle pole localization were partially but not completely rescued (Fig. 5c, d), indicating that the mitotic regulation reduced FUSN’s ability^42^. Moreover, both GFP-NuMA and GFP-NuMA-S1969D were similar in phase separation and spindle pole localization in cells no matter with or without nocodazole treatment, whereas S1969A showed an enhancement in phase separation and spindle pole localization. Next, we screened NuMA-binding proteins by mass spectrometry (MS; Supplementary Data 1), and found that KifC1, a known minus-end-directed kinesin motor protein^43^, is a NuMA-binding protein. Via IF and immunoprecipitation (IP), we found that KifC1 was enriched in NuMA droplets situated on the microtubules of the assembling spindle and nocodazole-treated droplets and that NuMA interacted with KifC1 in early mitosis (Fig. 5e–g and Supplementary Fig. 6b). When KifC1 was knocked down, the enrichment of NuMA on the poles was significantly reduced (Fig. 5h–j). In another way, when NuMA was depleted with IAA, the localization of KifC1 on the spindle poles was also reduced but its spindle microtubule localization was not affected (Supplementary Fig. 6c, d), indicating that the spindle microtubule localization of KifC1 is independent of NuMA.

Taken together, we conclude that NuMA phase separation is responsible for its localization on spindle poles through its aa1911–1925 motif in CT and that KifC1 facilitates the localization of NuMA on spindle poles.

### NuMA droplets enrich Kif2A for microtubule flux

**Fig. 6:**
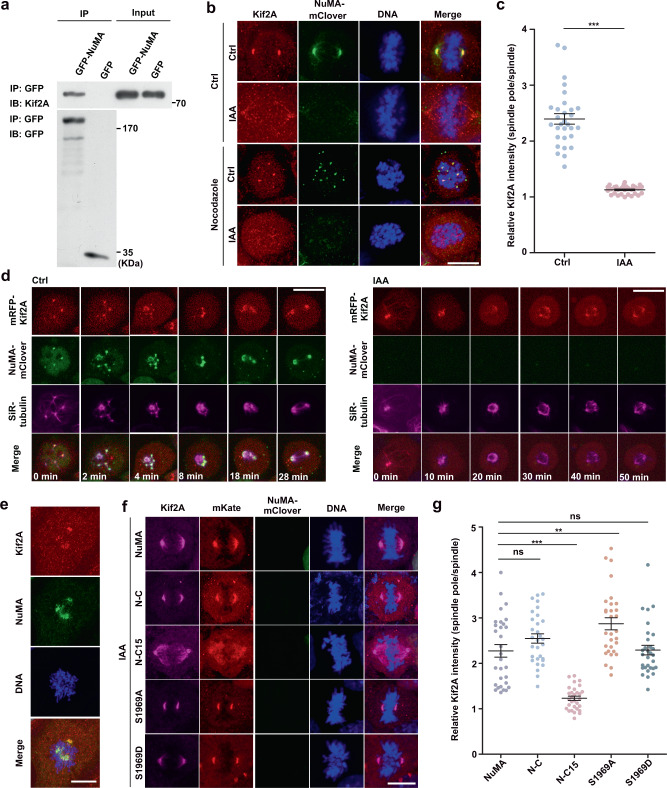
NuMA concentrates Kif2A to promote microtubule flux. **a** Immunoprecipitation assay showing that NuMA interacts with Kif2A. HEK293T cells were transfected with GFP, GFP-NuMA, arrested in mitosis with nocodazole, and processed for IP assay with GFP-Trap beads. **b** Immunofluorescence images of mitotic spindles upon NuMA depletion in the control and nocodazole-treated NuMA-mACF cells, followed by staining with anti-Kif2A antibody and DAPI. **c** Quantification of the relative fluorescence intensity of Kif2A on the spindle poles (mean ± SEM, *n* = 30 cells in 1 experiment, 3 independent experiments were repeated). The method of measurement was mentioned in Fig. 5d. Unpaired two-tailed *t* test: *P* < 0.0001 for Ctrl/IAA. **d** Time-lapse images showing the dynamic process of exogenous Kif2A in the presence of NuMA (Ctrl) or in NuMA-depleted (IAA) cells. Images were collected every 2 min. Microtubules were probed by SiR-tubulin (purple). **e** Immunofluorescence images of mitotic HeLa cells stained with anti-NuMA (green) and Kif2A (red) antibodies. **f** Immunofluorescence images of mitotic spindles in endogenous NuMA-depleted NuMA-mACF cells with stable expression of exogenous mKate-NuMA, mKate-N-C, mKate-N-C15, mKate-S1969A, or mKate-S1969D, followed by staining with anti-Kif2A antibody and DAPI for DNA. **g** Quantification of relative Kif2A fluorescence intensity on spindle poles in NuMA-mACF cells treated as in **f** (mean ± SEM, *n* = 30 cells in 1 experiment, 3 independent experiments were repeated). Unpaired two-tailed *t* test: *P* = 0.1177 for NuMA/N-C, *P* < 0.0001 for NuMA/N-C15, *P* = 0.0028 for NuMA/S1969A, *P* = 0.9229 for NuMA/S1969D. **P < 0.01, and ***P < 0.001, ns, not significant. Scale bars, 10 μm.

The dynamic balance between microtubule polymerization and depolymerization maintains the dynamic structure and length of the mitotic spindle during its assembly^7,8,10^. In determining how NuMA regulates the structural dynamics and length control of the mitotic spindle, we also screened NuMA-binding proteins by MS and identified that Kif2A, which depolymerizes microtubules on spindle poles, was also a NuMA-binding partner, whereas other known polymerases and depolymerases were not found with NuMA by MS (Supplementary Data 1). Through reciprocal IP assays and IF, we not only confirmed that NuMA interacted with Kif2A in mitotic cells (Fig. 6a and Supplementary 7a) but also found that Kif2A was enriched in NuMA droplets regardless of treatment with or without nocodazole, and this enrichment in NuMA droplets was abolished by NuMA depletion (Fig. 6b, c). In mitotic cells, Kif2A presented a highly consistent colocalization with NuMA on the spindle poles, and when endogenous NuMA was depleted, Kif2A was also removed from the spindle poles and dispersed into the cytoplasm (Fig. 6b, c). Then we co-depleted Kif2A and NuMA in HeLa cells and measured the rates of microtubule flux. We found that microtubule flux was reduced no matter with or without NuMA, indicating that Kif2A depolymerizes microtubules to maintain the correct spindle length (Supplementary Fig. 7b). In contrast, Kif2A RNAi knockdown did not disturb the spindle pole localization of NuMA (Supplementary Fig. 7c–e). Next, we explored the dynamic localization behaviors of NuMA and Kif2A during mitotic entry. In the presence of NuMA, Kif2A localized to NuMA droplets during NEBD and was transported to the spindle poles. When bipolar spindle was established, Kif2A were sequestered onto the spindle poles. When NuMA was deleted, Kif2A still could bind with microtubules in prometaphase, whereas Kif2A could not concentrate on spindle poles but dispersed into the whole spindle in metaphase, indicating that NuMA would not mediate the interaction between Kif2A and microtubules but sequestered Kif2A on spindle poles in metaphase (Fig. 6d and Supplementary Movies 12 and 13). Then IF images also suggested that endogenous Kif2A was recruited to endogenous NuMA droplets in prophase (Fig. 6e). Through stably expressing NuMA truncation mutants in endogenous NuMA-depleted cell lines, we found that expression of wild-type, N-C, and S1969D, all of which possess phase separation ability, rescued the localization of Kif2A on the spindle poles at approximately the same level as that in cells without endogenous NuMA depletion (Fig. 6f, g). In contrast, expressing N-C15 failed to rescue the normal spindle pole localization of Kif2A; and more importantly, expressing S1969A, which showed strong phase separation and enhanced spindle pole localization, recruited more Kif2A to the spindle poles.

To understand the underlying molecular mechanisms, through IP assay using mitotic HEK293T cells expressing truncated GFP-tagged NuMA proteins, we further determined the interactions of NuMA proteins with Kif2A. We observed that, while both NT and CC did not interact with Kif2A, CT weakly interacted with and N-C strongly with Kif2A (Supplementary Fig. 7f), consistent with their abilities in phase separation (Figs. 3a and 5c). Interestingly, N-C15, which could not phase-separate in mitosis (Fig. 5c), still interacted with Kif2A (Supplementary Fig. 7f), indicating that the 15 amino acids only reduced the ability of phase separation but do not affect its interaction with Kif2A.

Thus, we conclude that Kif2A is a passenger protein in NuMA droplets and that the phase-separated NuMA recruits Kif2A to the mitotic spindle poles to regulate the poleward microtubule flux of the mitotic spindle.

### Disrupting phase separation elongates spindle length

**Fig. 7:**
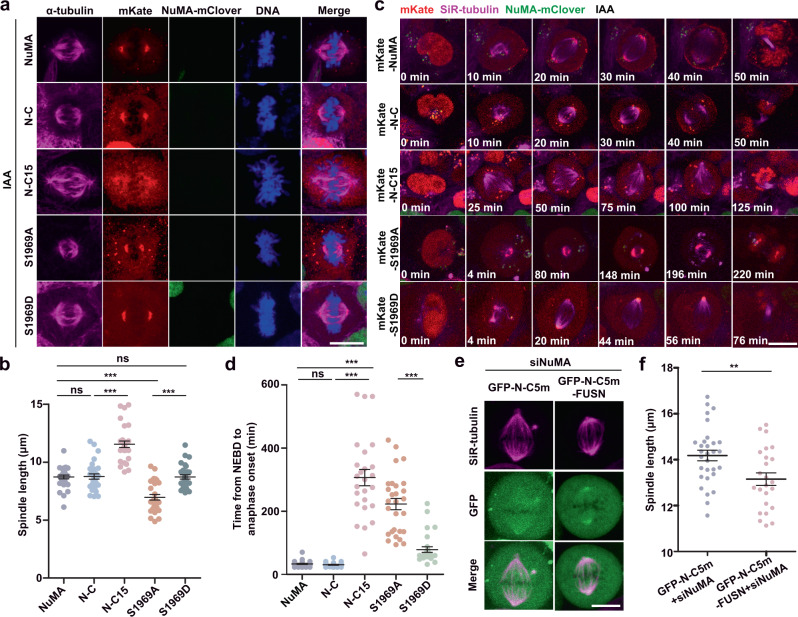
Disrupting phase separation elongates spindle length. **a** Immunofluorescence images of mitotic spindles in endogenous NuMA-depleted NuMA-mACF cells stably expressing exogenous mKate-NuMA, mKate-N-C, mKate-N-C15, mKate-S1969A, and mKate-S1969D, followed by staining with anti-α-tubulin antibody and DAPI for DNA. **b** Quantification of spindle length in endogenous NuMA-depleted NuMA-mACF cells in **a** (mean ± SEM, *n* = 30 cells in 1 experiment, 3 independent experiments were repeated). Unpaired two-tailed *t* test: *P* = 0.8827 for NuMA/N-C, *P* < 0.0001 for N-C/N-C15, *P* < 0.0001 for NuMA/S1969A, *P* = 0.9691 for NuMA/S1969D, *P* < 0.0001 for NuMA-S1969A/NuMA-S1969D. **c** Time lapse of cell images showing the mitotic progress after rescuing different truncated mutants of NuMA upon endogenous NuMA depletion. **d** Quantification of the time from NEBD to anaphase onset of cells treated as in **c** (mean ± SEM, *n* = 30, 24, 25, 28, and 28 cells were measured for NuMA, N-C, N-C15, S1969A, and S1969D, respectively). Unpaired two-tailed *t* test: *P* = 0.2386 for NuMA/N-C, *P* < 0.0001 for N-C/N-C15, *P* < 0.0001 for NuMA/S1969A, *P* < 0.0001 for NuMA-S1969A/NuMA-S1969D. **e** Live cell images of mitotic HeLa cells expressed with GFP-N-C5m or GFP-N-C5m-FUSN after NuMA depletion. Microtubules were probed with SiR-tubulin. **f** Quantification of spindle length in **e** (mean ± SEM, *n* = 30, 24 cells were measured for N-C5m and N-C5m-FUSN in 1 experiment, 3 independent experiments were repeated). Unpaired two-tailed *t* test: *P* = 0.0055 for N-C5m/N-C5m-FUSN. **P < 0.01, and ***P < 0.001, ns, not significant. Scale bars, 10 μm.

Finally, we determined the functions of the spindle pole recruitment of Kif2A via NuMA phase separation. Via expressing NuMA mutants in endogenous NuMA-depleted cells, we found that both the wild-type and the N-C mutant were located at the spindle poles and they could fully rescue the defects in morphology and length of the mitotic spindle induced by NuMA depletion; and in contrast, expressing the phase separation-incapable mutant N-C15 failed to rescue the defects, resulting in significantly longer spindle formation than expressing the wild type and N-C (Fig. 7a, b). More interestingly, expressing the unphosphorylated mutant S1969A resulted in shorter spindle formation than expressing the wild type, N-C, or N-C15, whereas expressing the phosphorylation-mimicking mutant S1969D resulted in relatively normal spindle assembly with similar length to expressing the wild type. Along with their expression, both the wild type and N-C gradually relocated from the interphase nucleus to the mitotic spindle and poles and successfully rescued the NuMA depletion-induced mitosis defects; and in contrast, the expressed N-C15 failed to relocate from the interphase nucleus to the mitotic spindle poles and was unable to rescue the mitosis defects (Fig. 7c, d). Moreover, S1969A expression in NuMA-depleted cells resulted in short spindle formation and strongly disrupted proper mitotic progression, whereas S1969D almost rescued the NuMA depletion-induced mitotic deficiency (Fig. 7c, d). We also overexpressed N-C5m and N-C5m-FUSN in HeLa cells after NuMA depletion. The results showed that, while N-C5m could not rescue the elongated spindle defects, N-C5m-FUSN, in which FUSN enhances phase separation of N-C5m, located to the spindle poles and partially rescued these defects (Fig. 7e, f). In addition, using 5% 1,6-hexanediol treatment to disrupt exogenous GFP-NuMA phase separation, we found that metaphase spindle length was quickly and efficiently elongated (Supplementary Fig. 8a, b and Supplementary Movies 14). In all, 5% 1,6-hexanediol treatment also significantly reduced phase separation of GFP-S1969A and GFP-S1969D in mitotic HeLa cells and resulted in a maximum elongation of the metaphase spindles (Supplementary Fig. 8c–f and Supplementary Movies 15 and 16). Thus, we conclude that the phase separation of NuMA is a key factor that controls the mitotic spindle assembly, the structural dynamics, and the metaphase spindle length by concentrating Kif2A at the spindle poles to regulate the poleward spindle microtubule flux.

## Discussion

The mitotic spindle is mainly composed of microtubules and diverse motor and microtubule-associated proteins and is highly dynamic with assembly, dynamic structural maintenance, and disassembly for faithful chromosome congression and segregation during the cell division. In this work, we discovered a previously unknown mechanism under which NuMA regulates the mitotic spindle assembly and structural dynamics via phase separation. Through phase separation, NuMA directly binds and recruits Kif2A to the spindle poles to depolymerize spindle microtubules and promote poleward microtubule flux for mitotic spindle assembly, structural dynamics, and function. Movement of NuMA to spindle poles is not an even procedure, but instead, is through poleward movement of phase-separated droplets along spindle microtubules. The phase-separated NuMA droplets also concentrate tubulins and sort microtubule asters into assembling spindle microtubule array for the mitotic spindle assembly and dynamics.

A well-assembled functional metaphase spindle is partitioned to distinct regions with two poles that focus spindle microtubules at the minus end and with the large spindle microtubule array that connects with the kinetochores of the chromosomes at the plus end on the equatorial plate. Once well assembled, the spindle maintains its morphology, structure, and length through dynamics of microtubule polymerization and depolymerization cycle under tight regulations until metaphase–anaphase transition occurs. Obviously, the spindle pole is one of the key regulation centers that not only focuses minus-end microtubules but also dynamically enriches key regulators and hosts multiple biochemical reactions to depolymerize the spindle microtubules to keep the poleward microtubule flux for dynamic balance of the spindle^8,10,44,45^. However, the underlying mechanisms for poleward movement of the spatiotemporal components and organization of the poles are still poorly understood. Here we identify that an abundant nuclear protein NuMA plays a crucial role at the spindle poles for spindle assembly, structural dynamics, and function through cell cycle-regulated phase separation. This work provides not only a previously uncovered principle of the bipolar spindle assembly and dynamics regulated by phase separation of NuMA and enrichment of key regulators for poleward spindle microtubule flux by NuMA droplets but also an important clue to study insight of the mechanisms for accurate chromosome congression and segregation for faithful cell proliferation.

Phase separation and translocation of NuMA to the mitotic spindle poles not only facilitates organization and focusing of the spindle microtubules to the poles but also enriches key regulators to the spindle poles for spindle assembly, structural dynamics, and function. One of the enriched key regulators is Kif2A, which directly catalyzes spindle microtubule depolymerization at the poles promoting the poleward spindle microtubule flux for the mitotic spindle assembly and structural dynamics. Consistently, in cells with no centrosomes, NuMA may organize formation of monopole- or bundle-like microtubule structures and bipolar mitotic spindle assembly in cooperation with other motor proteins^35^. In contrast to somatic cells, the acentrosomal mammalian oocytes use a phase separation-formed liquid-like spindle domain, consisting of centrosomal proteins, centriolar satellite proteins, minus-end binding proteins, and dynein-related proteins but not NuMA, to control microtubule nucleation and stability and acentrosomal bipolar spindle assembly^29^. In our work, we use somatic cells and discover that NuMA plays a central role in regulating the spindle assembly and structural dynamics through phase separation to concentrate numerous key microtubule regulators to the spindle poles.

**Fig. 8:**
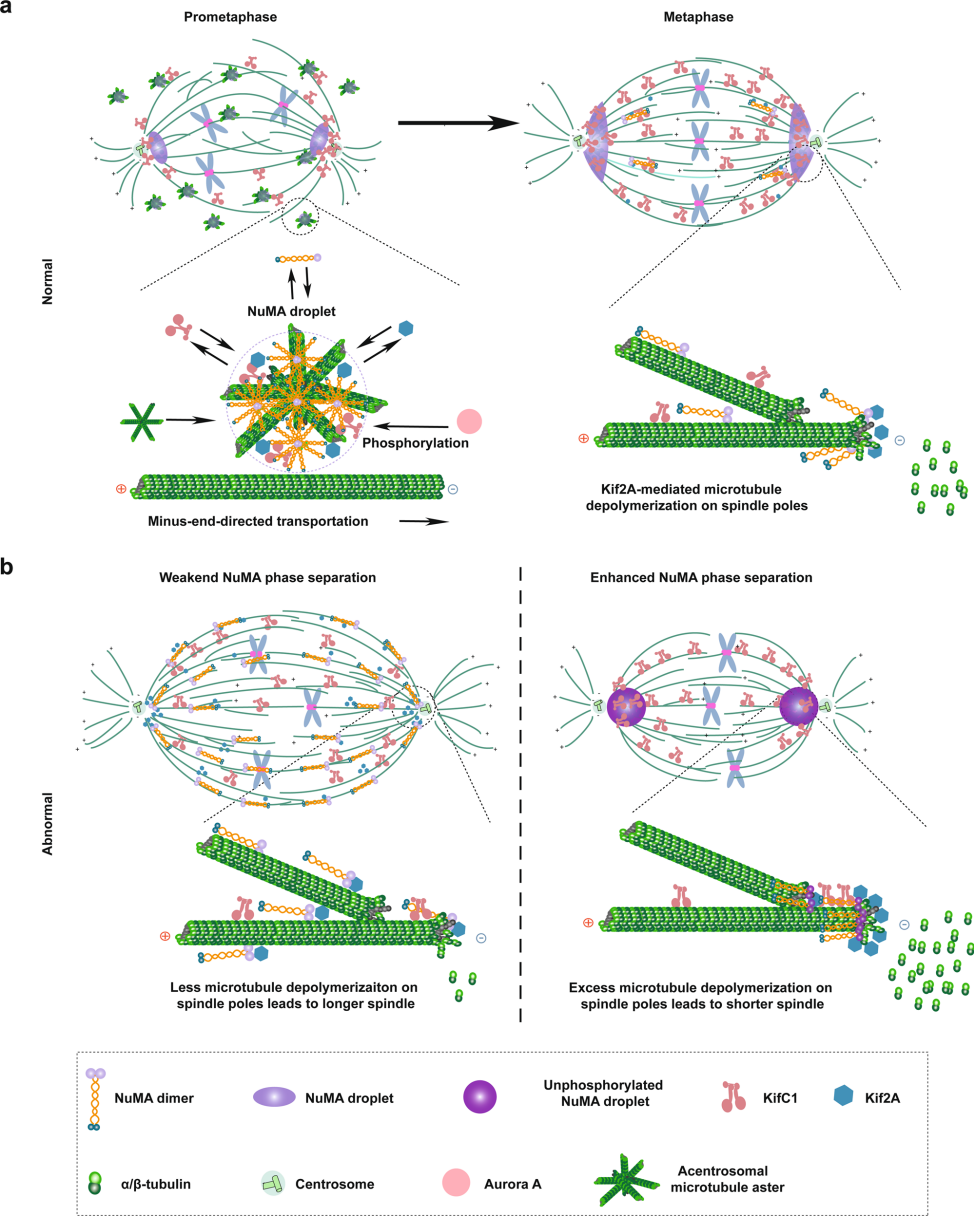
A working model elucidating that NuMA phase separation regulates spindle dynamics. **a** During mitotic entry, Aurora A phosphorylates and promotes NuMA to form mitotic liquid-like droplets through phase separation, which then concentrate tubulin and bind with Kif2A and KifC1 and acentrosomal microtubule asters. Along with cell cycle progression and capture by spindle microtubules, these NuMA droplets sort acentrosomal microtubule asters into the spindle microtubule mass. Until metaphase, the phase-separated NuMA dynamically sequesters key spindle assembly regulators, including Kif2A, which then depolymerizes spindle microtubules to maintain steady spindle dynamics. **b** Disruption of NuMA phase separation results in dispersion of NuMA on the whole spindle but not to the poles, leading to reduction of microtubule depolymerization on spindle poles and elongated spindle formation due to failure of Kif2A concentration at the spindle poles. Alternatively, increasing the phase separation ability of NuMA, such as inhibiting phosphorylation by Aurora A, displays an enhanced phase separation ability and weakened dynamics of phase-separated NuMA droplets, leading to excess Kif2A on the spindle poles to depolymerize spindle microtubules and shorten the mitotic spindle.

Based on our present work, we propose a working model to illustrate the role of NuMA in regulating the mitotic spindle assembly, structural dynamics, and function through phase separation (Fig. 8). We propose that, under the cell cycle control, NuMA initiates phase separation in the nucleus during the mitotic entry in a microtubule-independent way. Along with NEBD, the phase-separated NuMA droplets are poleward transported to the mitotic spindle poles along spindle microtubules. KifC1, a known minus-end-directed kinesin motor protein, may facilitate this process. During this poleward transportation process, these NuMA droplets concentrate tubulin proteins and sort microtubule asters into the spindle microtubule mass and enrich key regulators to the spindle poles for the mitotic spindle assembly, dynamic structural maintenance and function. One of the key enriched regulators is Kif2A, which then depolymerizes the spindle microtubules and hence regulates the poleward spindle microtubule flux that is an essential process for mitotic spindle assembly, structural dynamics, and function. Phase separation of NuMA is carried out by its C-terminus in a regulated way, in which aa1911–1925 motif plays a crucial role. According to sequence analysis, CC and CT are both disordered regions. However, only CT could phase-separate and no phase separation is observed for CC in all the conditions tested. Considering that CT could oligomerize in vitro and oligomerization contributes more than disordered regions to driving LLPS in other systems^37,46^, we suggest that the oligomerization of CT is mainly responsible for NuMA phase separation. Due to the oligomerization of CT, the peak shape of the analytical size-exclusion chromatography is asymmetric in 670 kD, which indicates that the purified proteins consist of not only large aggregates but also monomers and oligomers in unsalted buffer. Phase-separated status of NuMA during interphase to mitosis transition is regulated at least in part by Aurora A that phosphorylates NuMA at S1969 residue and fluidizes the NuMA droplets. S1969 phosphorylation of NuMA not only contributes to its proper spindle pole localization but also promotes its outward relocation to other regions such as to the cell cortex, where it crosslinks microtubules with the cell cortex and controls the spindle orientation. Unphosphorylated NuMA concentrates on the spindle poles, where it may sequester more Kif2A, whereas S1969-phsophorylated NuMA may be transported elsewhere for local functions. Disrupting phase separation will lengthen the mitotic spindle, no matter whether the cell expresses wild-type, S1969-phosphorylated, or S1969-unphosphorylated NuMA, indicating the importance of NuMA phase separation. In conclusion, this work discovers a previously unknown molecular mechanism by which NuMA orchestrates the mitotic spindle assembly, dynamic spindle maintenance, and function, and this work may provide insights for understanding the fundamental issues of the cell life, especially of the cell division and proliferation processes.

## Methods

### Plasmid construction, antibody preparation, chemicals, and recombinant proteins

GFP-NuMA constructs were generated by cloning human NuMA1 from pEYFP-C1-NuMA (from the J. Zhou laboratory, Nankai University, China). NuMA-mCherry-Cry2WT was generated by cloning human NuMA1 into pHR-mCherry-Cry2 (from the Xiong Ji laboratory, Peking University, China). The His-GFP-CTD constructs were generated by cloning GFP-NuMA into pET28a. NuMA-mKate was generated by cloning human NuMA1 into pmKate-N. Other truncations and mutants of NuMA were generated by PCR with specific primers. The GFP-KifC1 and GFP-Kif2A constructs were from our laboratory. All antibodies used in this study were as follows: rabbit anti-NuMA (Abcam, ab97585, 1:250 for IF, 1:1000 for western blotting (WB)), mouse anti-NuMA (Santa Cruz Biotechnology, sc-365532, 1:1000 for WB), mouse anti-α-tubulin (Sigma, T9026, 1:500 for IF, 1:1500 for WB), mouse anti-Kif2A (Santa Cruz Biotechnology, sc-271471, 1:200 for IF, 1:1000 for WB), mouse anti-KifC1 (in-house generation, 1:100 for IF, 1:500 for WB), mouse anti-pT288-Aurora A (Cell Signaling Technology, C39D8, 1:200 for IF), rabbit anti-p-Aurora A/B/C (Cell Signaling Technology, 2914S, 1:1000 for WB), mouse anti-GFP (Abmart, M20004, 1:5000 for WB), mouse anti-GAPDH (Proteintech, 60004-1-lg, 1:5000 for WB), rabbit anti-γ-tubulin (Sigma-Aldrich, T3559, 1:500 for IF), mouse anti-IgG Alexa Fluor 488 (Invitrogen, A-21202, 1:200 for IF), rabbit anti-IgG Alexa Fluor 488 (Invitrogen, A-21206, 1:200 for IF), mouse anti-IgG Alexa Fluor 594 (Invitrogen, A-21203, 1:200 for IF), rabbit anti-IgG Alexa Fluor 594 (Invitrogen, A-21207, 1:200 for IF), mouse anti-IgG Alexa Fluor 647 (Invitrogen, A32728, 1:200 for IF), rabbit anti-IgG Alexa Fluor 647 (Invitrogen, A-31573, 1:200 for IF). IAA (BioRuler, RH30145), Dox (Qualityard, QYR4881), MLN8237 (Selleck, S1133), nocodazole (Sigma-Aldrich, M1404), 1,6-hexanediol (Sigma-Aldrich, 240117-50G), PEG3350 (Sigma-Aldrich, P-3640), Dulbecco’s modified Eagle’s medium (DMEM; Gibco, C11995500BT), TurboFect (Gibco, R0531), fetal bovine serum (CellMax, SA10), and recombinant human Aurora A protein (Active) (Abcam, ab268355). All animal experiments for raising antibodies were performed in the Laboratory Animal Center of Peking University in accordance with the National Institute of Health Guide for Care and Use of Laboratory Animals according to guidelines approved by the Institutional Animal Care and Use Committee at Peking University.

### Cell culture, cell line generation, transfection, and synchronization

HeLa and HEK293T cells were obtained from American Type Culture Collection (ATCC, CCL-2, CRL-11268). The HCT116 TetOsTIR1 NuMA–mAID–mClover–3X–FLAG (NuMA-mACF) cell line was provided by Dr. Tomomi Kiyomitsu (Nagoya University, Japan). Related NuMA-mACF cell lines stably expressing NuMA truncated mutants were generated by lentivirus infection and screened by fluorescence-activated cell sorting (FACS). Stable RFP-H2B-expressing cell lines were generated by screening single colonies using 500 μg/ml G418 after transfection with RFP-H2B plasmids in HeLa cells. Stable GFP-NuMA-expressing HeLa cells were screened by FACS after transfection with lentiviruses. HCT116 cells were cultured in McCoy’s 5A medium (Invitrogen), and other cell lines were cultured in DMEM (Gibco), both supplemented with 10% (v/v) bovine calf serum at 37 °C in a 5% CO_2_ atmosphere. To synchronize to metaphase for IF analysis, HeLa cells were treated with thymidine for 17–24 h, released for 9 h, and then incubated with 10 μM MG132 for 1 h to fully establish the bipolar spindle. To synchronize mitosis for IP, HEK293T cells were treated with thymidine for 20 h, released for 3 h, and then supplemented with 100 ng/ml nocodazole for another 10 h. To degrade endogenous NuMA-mClover, NuMA-mACF cells were treated with 1 μg/ml Dox and 500 μM IAA for 24 h. For plasmid and RNA transfection in HeLa and HEK293T cells, Turbofect (Invitrogen) and PEI were used according to the manufacturer’s instructions. The following small interfering RNAs (siRNAs) were synthesized by GenePharma:

NuMA siRNA (5′-GGCGUGGCAGGAGAAGUUCUU-3′),

Kif2A siRNA (5′-GGCAAAGAGAUUGACCUGG-3′),

KifC1 siRNA-1 (5′-CCUCAACUCUCUACGCUUUTT-3′),

KifC1 siRNA-2 (5′-GCCCAGAAUGAACGGUCAUTT-3′).

### Protein expression and purification

To obtain soluble recombinant proteins, GFP-CT was inserted into the pET28a vector and expressed in *Escherichia coli* BL21 cells (DE3 pLys), which were induced with 0.2 mM IPTG for 18 h at 16 °C. Cell pellets were collected and resuspended in unsalted lysis buffer (50 mM Tris-HCl, pH 8.0, 20% glycerol) with protease inhibitors (1 mM phenylmethylsulfonyl fluoride and protease mixture inhibitor). Cells were sonicated and then clarified by centrifugation for 20 min at 146,000 × *g*.

To purify constructs, the clarified lysate was incubated with Ni-NTA agarose (QIAGEN) for 1 h. The agarose beads were washed with 10 column volumes of wash buffer (50 mM Tris-HCl, pH 8.0, 20% glycerol, 40 mM imidazole), and the protein was eluted with 400 mM imidazole. Proteins were then aliquoted in PCR tubes, flash-frozen in liquid nitrogen, and stored at −80 °C. Protein concentration was determined by measuring absorbance at 280 nm using a NanoDropND-1000 spectrophotometer (Thermo Scientific).

### Size-exclusion chromatography

To determine their status in solution, 5 mg purified GFP-CT proteins were loaded onto Superdex 200 Increase (GE Healthcare) connected to ÄKTA Pure (GE Healthcare). Samples were eluted in a buffer [50 mM Tris-HCl (pH 8.0)] at the flow rate of 0.5 ml/min for 60 min. The data were collected and analyzed by the Unicorn 7 software (GE Healthcare). An equal amount of Gel Filtration Standard (Bio-rad) was analyzed under the same conditions as a marker.

### Phase separation assay and FRAP

NuMA droplets were formed by adding concentrated GFP-CT protein to phase separation buffer (50 mM Tris-HCl, pH 8.0, 10% glycerol, 150 mM KCl) containing polyethylene glycol in a slide chamber. For most experiments, NuMA condensates were visualized, and images were also taken with a confocal microscope. FRAP experiments were performed on a spinning disk microscope with a ×60 oil objective. NuMA droplets (in vivo and in vitro) were bleached for 50 cycles using a laser intensity of 80% at 480 nm (for GFP) or 561 nm (for mCherry). Recovery was recorded for the indicated time. The fluorescence intensity of the photobleached area was normalized to the intensity of the unbleached area.

### IF microscopy and live-cell imaging

For live-cell imaging, cells were plated on a glass-bottom dish. Before imaging, the dishes were locked in a heated chamber (37 °C) supplemented with 5% CO_2_. Images were acquired using PerkinElmer UltraView VoX spinning disk confocal microscope or Andor Dragonfly spinning disk confocal microscope with a ×60/1.4 NA oil objective lens of EMCCD followed by processing in the Imaris 9.5 and Volocity 6.1.1 softwares. For IF imaging, cells were grown on coverslips and fixed in precooled methanol for 5 min on ice followed by incubation with primary antibodies (diluted in phosphate-buffered saline (PBS) containing 3% bovine serum albumin) overnight at 4 °C. After three washes in PBS, the cells were incubated with secondary antibodies for 1 h at room temperature. Coverslips were mounted with Mowiol containing 1 μg/ml 4,6-diamidino-2-phenylindole and analyzed on a DeltaVision imaging system (Applied Precision) equipped with an Olympus IX-71 inverted microscope and ×100/1.4 NA oil objective lens. The images were captured by a Cool-Snap HQ2 CCD camera. All IF images were processed for maximum intensity projection.

### Immunoprecipitation

Mitotic HEK293T cells transfected with the indicated constructs were collected and lysed on ice in lysis buffer (20 mM Tris-HCl, pH 8.0, 150 mM NaCl, 2 mM EGTA, 0.5 mM EDTA, 0.5% NP-40, 5 mM NaF, 1 mM Na_3_VO_4_, 1 mM phenylmethylsulfonyl fluoride, and protease mixture inhibitor) for 30 min. Lysates were centrifuged at 15,000 × *g* for 15 min, and supernatants were incubated with beads conjugated with GFP-Trap (Chromo Tek, gtc-20) for 1.5 h at 4 °C. After five washes with lysis buffer, the beads were suspended in gel sample buffer, and the bound proteins were analyzed by WB.

### Cytosol preparation

HeLa cells were synchronized to interphase and mitosis with thymidine and nocodazole. The cells were centrifuged for 5 min at 1000 × *g* and then washed and resuspended in an equal volume of KHM buffer (78 mM KCI, 50 mM Hepes-KOH pH 7.0, 4 mM MgCl_2_, 10 mM EGTA, 8.37 mM CaCl_2_, 1 mM dithiothreitol, 20 pM cytochalasin B). Harvested cells were homogenized with a homogenizer. The lysates were centrifuged at 160,000 × *g* for 1 h, and supernatant fractions were collected as cytosol.

### GFP-α-tubulin photoactivation analysis

HeLa cells were transfected with PA GFP-α-tubulin and synchronized to mitosis. A 405 nm laser was used to activate GFP-α-tubulin in a rectangular region near the microtubule plus ends inside the spindle. Imaging was performed with a spinning disc confocal microscope equipped with an inverted microscope (Nikon TiE) and a ×60/1.4 NA oil objective lenses and images were acquired every 10 s. To analyze the velocity of microtubule flux, the quantification of fluorescence intensity of the activated areas and the distance of fluorescent signal movement were analyzed using the Volocity 6.1.1 software.

### MS analysis

Synchronously growing HEK293T cells transfected with GFP-NuMA were subjected to IP with GFP-Trap beads. The resulting immunoprecipitates were separated by 8% sodium dodecyl sulfate-polyacrylamide gel electrophoresis gels. The proteins on the gels were stained with Coomassie Brilliant Blue, digested with trypsin, and analyzed on a Q Exactive Plus mass spectrometer (Thermo Fisher Scientific) for NuMA-interacting proteins. The MS data were alignment with Mouse Reviewed Swiss-Port database by the Proteome Discoverer 2.2 software.

### Microtubule-binding assay in vitro

The assay to make Taxol-stabilized microtubules was described previously^28^. Two microliters of Cushion Buffer (80 mM PIPES, 2 mM MgCl_2_, 0.5 mM EGTA, 40% glycerol, pH 6.9) containing 1 mM GTP was added to 2 μl of the tubulin stock to obtain a final concentration of 18 μM tubulin. The mixture was incubated at 35 °C for 15 min and then diluted with 100 μl of prewarmed (RT) General Tubulin Buffer (80 mM PIPES, 2 mM MgCl_2_, 0.5 mM EGTA, pH 7.0). Taxol was added to this mixture to a final concentration of 20 μM. The mixture was layered onto 400 μl of Cushion Buffer containing 20 μM Taxol and centrifuged at 100,000 × *g* at 25 °C for 30 min to separate the polymerized microtubules from the tubulin. After removing the supernatant, the pelleted microtubules were resuspended in 100 μl of General Tubulin Buffer containing 20 μM Taxol.

To observe the binding between GFP-CT and Taxol-stabilized microtubules in vitro, 3 μl of microtubules was mixed with GFP-CT at the mentioned concentration. After incubating the mixture at 37 °C for 5 min, 10 μl of the mixture was gently placed on chamber slides for microscopic analyses.

### Measurement of relative fluorescence intensity on spindle poles

Four square boxes of 3 × 3 μm were drawn, two on two spindle poles and the other two on their adjacent spindle areas. The mean fluorescence intensity was measured using the Velocity software (version 6.1.1). The relative fluorescence intensity was defined by the ratio of the mean intensity on spindle poles and on the adjacent spindle areas. When KifC1-depleted cells had multiple poles, the measurement was carried out only on half of the spindle with a single focused pole.

### Statistical and reproducibility

We used the Prism software (version 5.0; GraphPad) for statistical analyses using unpaired two-tailed *t* tests. All data are presented as the mean values ± SEM. *P* values in all graphs were generated with tests as indicated in figure legends and are represented as follows: ns, *P* > 0.05; **P < 0.01; and ***P < 0.001. For the representative experiments shown in figures, three independently experiments were repeated with similar results.

### Sequence analysis for protein disorder

The PONDR program (http://www.pondr.com/) was used to analyze disordered regions of NuMA. Two predictors (VL3, and VSL2) indicated that NuMA has a high disorder disposition.

### Reporting summary

Further information on research design is available in the Nature Research Reporting Summary linked to this article.

## Supplementary information

Supplementary Information

Description of Additional Supplementary Files

Supplementary Data 1

Supplementary Movie 1

Supplementary Movie 2

Supplementary Movie 3

Supplementary Movie 4

Supplementary Movie 5

Supplementary Movie 6

Supplementary Movie 7

Supplementary Movie 8

Supplementary Movie 9

Supplementary Movie 10

Supplementary Movie 11

Supplementary Movie 12

Supplementary Movie 13

Supplementary Movie 14

Supplementary Movie 15

Supplementary Movie 16

Reporting Summary

## Data Availability

All relevant data supporting the key findings of this study are available within the article, Supplementary Information and Source Data. Biological materials including cell lines and custom antibodies generated for this study will be shared upon request within the limits of respective material transfer agreements for as long as they are available in the laboratory. Source data are provided with this paper.

## Source data

Source Data
