# Supplementary material for: NuMA regulates mitotic spindle assembly, structural dynamics and function via phase separation
Source: Nat Commun. 2021 Dec 9;12:7157. doi: 10.1038/s41467-021-27528-6 (PMC8660824; doi:10.1038/s41467-021-27528-6)
Supplement: Supplementary file 1 — Supplementary Information [file 41467_2021_27528_MOESM1_ESM.pdf]

Supplementary Information

NuMA regulates mitotic spindle assembly, structural dynamics and function via phase separation

Mengjie Sun, Mingkang Jia, He Ren, Biying Yang, Wangfei Chi, Guangwei Xin, Qing Jiang, and Chuanmao Zhang*

Correspondence to: zhangcm@pku.edu.cn

This PDF file includes:

Supplementary Figures 1-8
Captions for Supplementary Figures 1-8

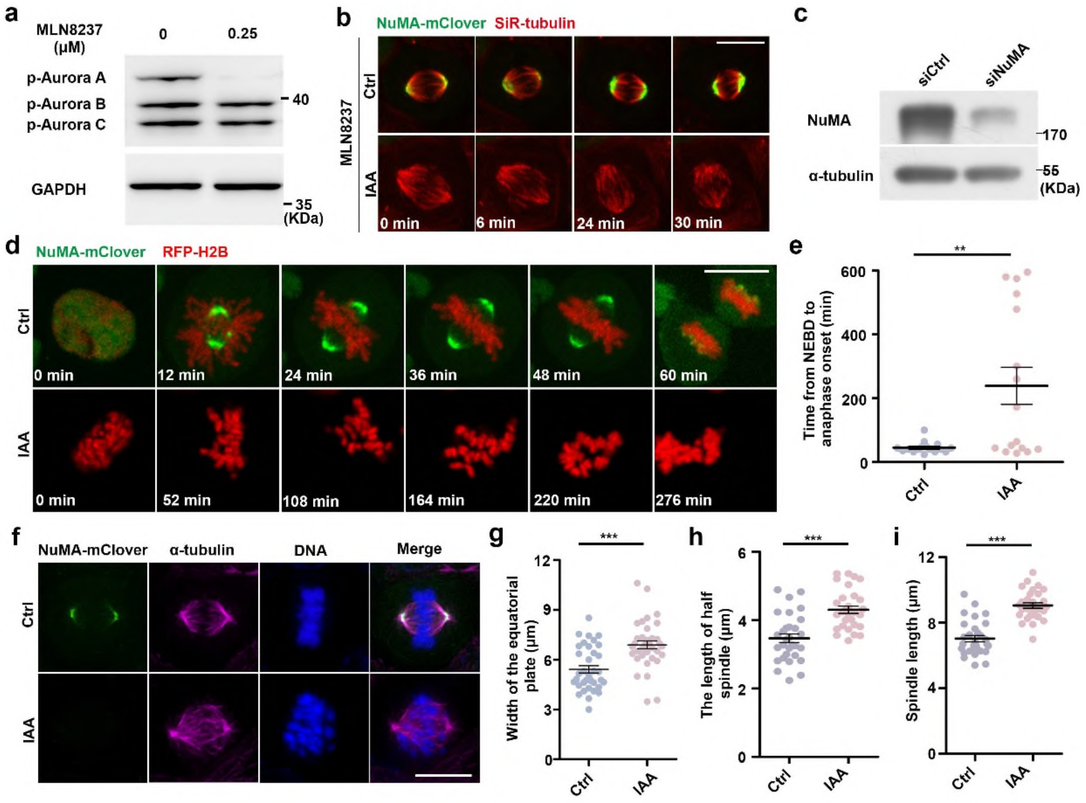

Supplementary Fig. 1 NuMA regulates spindle length, chromosome congression and segregation. **a** Immunoblotting for phospho-Aurora A/-B/-C and GAPDH expression of mitotic HeLa cells treated with control or 0.25 μM MLN8237. **b** Time-lapse images of mitotic spindles in MLN8237-treated NuMA-mACF cells with or without endogenous NuMA. Microtubules were probed with SiR-tubulin (red). **c** Immunoblotting for NuMA and α-tubulin expression in control and NuMA siRNA-treated HeLa cells. **d** Time-lapse images showing that NuMA depletion disturbs chromosome segregation. NuMA-mACF cells were transfected with RFP-H2B and treated with control or IAA. **e** Quantification of the time use from NEBD to anaphase onset in (**d**) (mean ± SEM, n=16 cells). Unpaired two-tailed t test: P=0.0024 for Ctrl/IAA. **f** Immunofluorescence images of mitotic spindles in control or IAA-treated NuMA-mACF cells, followed by staining with anti-α-tubulin antibody and DAPI for DNA. **g** Quantification of the spindle width at the equatorial plate (mean ± SEM, n=36 cells were shown here for one replicated experiment, three independent experiments were repeated). Unpaired two-tailed t test: P<0.0001 for Ctrl/IAA. **h** Quantification of the length of half spindles in control NuMA-mACF cells and NuMA-depleted cells

with widened equatorial plates (mean ± SEM, n=30 cells were shown here for one replicated experiment, three independent experiments were repeated). Unpaired two-tailed t test: P<0.0001 for Ctrl/IAA. **i** Quantification of spindle length in control NuMA-mACF cells and NuMA-depleted cells with normal equatorial plates (mean ± SEM, n=30 cells were shown here for one replicated experiment, three independent experiments were repeated). Unpaired two-tailed t test: P<0.0001 for Ctrl/IAA. *P < 0.05, **P < 0.01, and ***P < 0.001, ns, not significant. Scale bars, 10 μm.

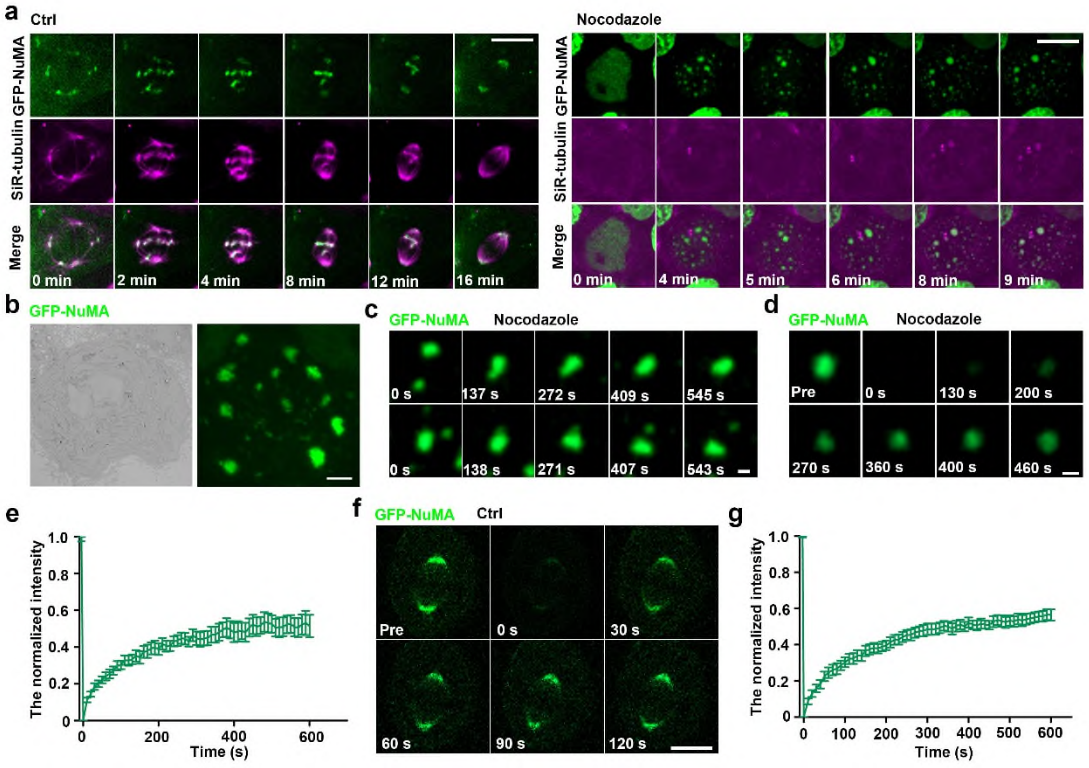

Supplementary Fig. 2 NuMA phase-separates in mitotic HeLa cells. a Time-lapse images showing NuMA forms droplets spontaneously along with NEBD in HeLa cells. HeLa cells stably expressing GFP-NuMA were treated without (Ctrl) or with (Nocodazole) nocodazole, and images were collected every 2 min. Microtubules were probed by SiR-tubulin (purple). **b** Correlative light electron microscopy (CL-EM) images of mitotic cells stably expressing GFP-NuMA with nocodazole treatment. Scale bar, 2 μm. **c** Time-lapse imaging showing fusion and fission of GFP-NuMA droplets in nocodazole-treated mitotic HeLa cells stably expressing GFP-NuMA. Scale bar, 1μm. **d, e** Time-lapse images (**d**) and quantification (**e**) of fluorescence recovery after

photobleaching (FRAP) of GFP-NuMA droplets after photobleaching in nocodazole-treated mitotic HeLa cells stably expressing GFP-NuMA (mean ± SEM, n=10 droplets). Scale bar, 2 μm. **f, g** Time-lapse images (**f**) and quantification (**g**) of FRAP of GFP-NuMA on the spindle pole after photobleaching in mitotic HeLa cells stably expressing GFP-NuMA (mean ± SEM, n=10 cells). Scale bars, 10 μm unless specified otherwise.

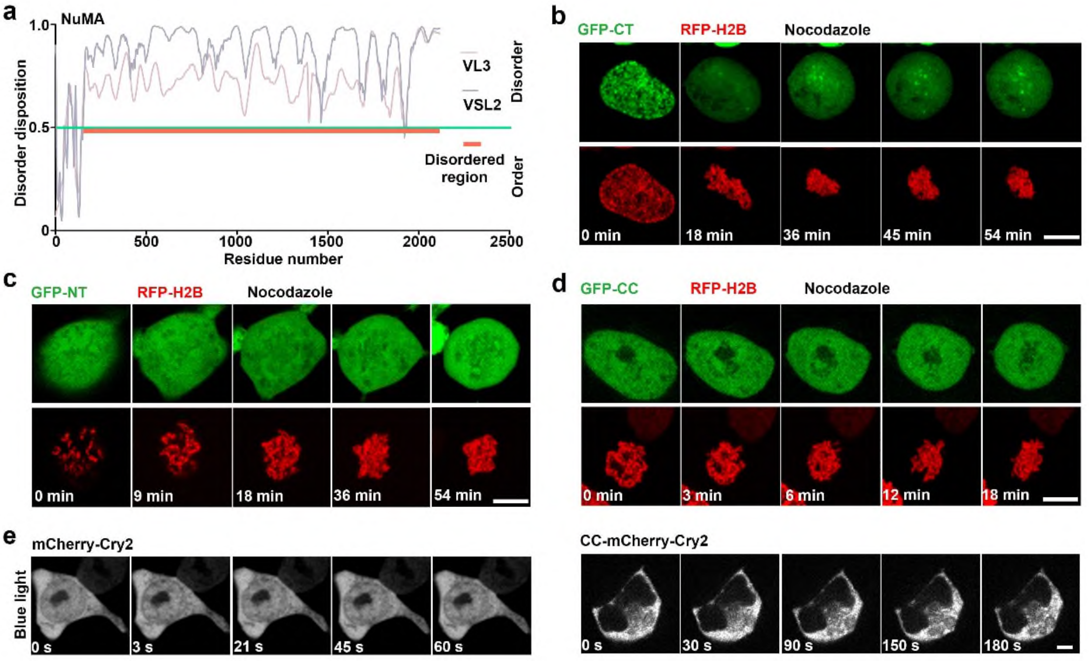

Supplementary Fig. 3 C-terminus is required for NuMA phase separation *in vivo*. **a,** Sequence features of NuMA. The line at 0.5 (y axis, green) is the cutoff for disorder (>0.5) and order (<0.5) predictions. VSL2 and VL3: predictors for disordered dispositions. The features were analyzed by PONDR. **b-d** Time-lapse microscopy images showing three NuMA domains with different behaviors during early mitosis. Nocodazole-treated HeLa cells stably expressing RFP-H2B were transfected with GFP-CT (**b**), GFP-NT (**c**) and GFP-CC (**d**), and images were collected every 3 min. Scale bars, 10 μm. **e,** Time-lapse microscopy images of HEK293T cells expressing either mCherry-Cry2 only or the coiled-coil domain (CC, aa213-1699). The cells were subjected to 488 nm laser excitation every 3 seconds for the indicated time, and images were collected by a 594 nm laser at the same time. Scale bars, 2 μm.

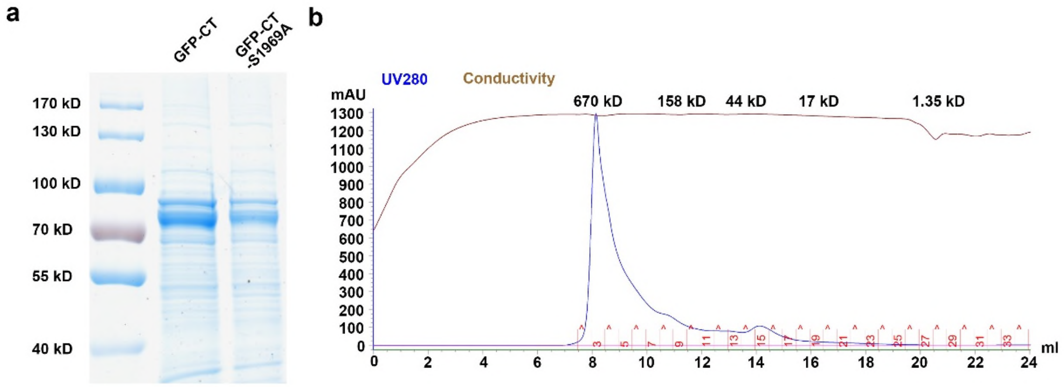

Supplementary Fig. 4 Purify GFP-CT and GFP-CT-S1969A proteins *in vitro*. a Coomassie Brilliant Blue–stained gel showing purified GFP-CT and GFP-CT-S1969A proteins. **b** Size-exclusion chromatography elution profiles of the GFP-CT proteins. The Gel Filtration Standard (Bio-rad) serves as a marker.

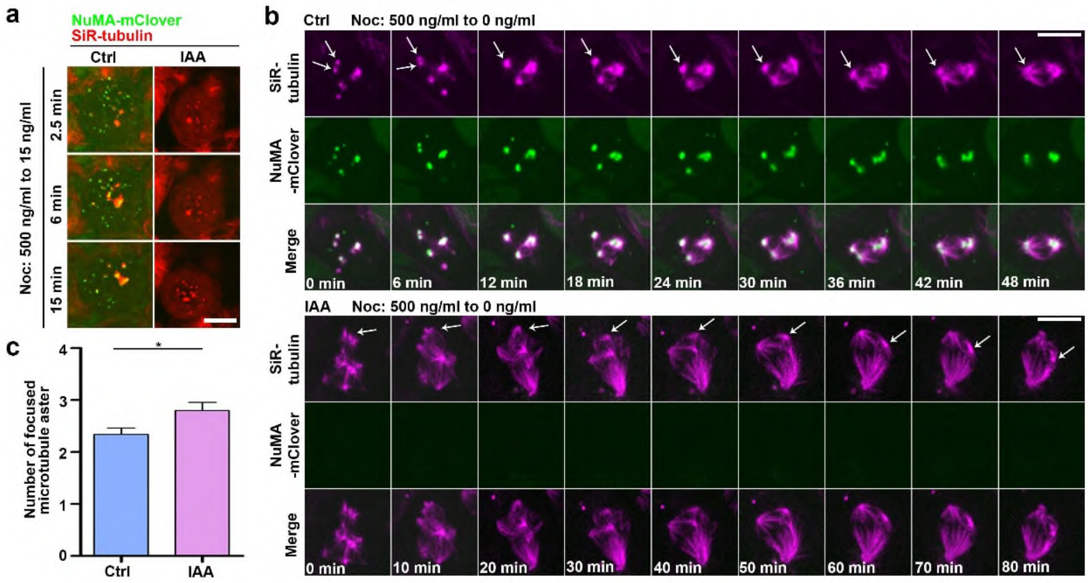

Supplementary Fig. 5 Phase-separated NuMA droplets sort microtubule asters into assembling mitotic spindles a Time-lapse microscopy images of 500 ng/ml nocodazole-arrested normal control or NuMA-depleted NuMA-mACF cells, followed by release into warmed fresh medium with 15 ng/ml nocodazole. Images were collected every 2.5 min. Microtubules were probed with SiR-tubulin (red). **b** Time-lapse microscopy images of 500 ng/ml nocodazole-arrested normal control or NuMA-depleted NuMA-mACF cells, followed by release into warmed fresh medium without

nocodazole. Images were collected every 2 min. Microtubules were probed with SiR-tubulin (purple). **c** Quantification of focused microtubule asters. NuMA-mACF cells were treated with 500 ng/ml nocodazole and then released into warmed fresh medium without nocodazole. The cells were fixed at 30 min. The number of microtubule asters/spindle poles at 30 min was counted (mean ± SEM, n=50 cells were measured per sample respectively). Unpaired two-tailed t test: P=0.0217 for Ctrl/IAA. *P < 0.05, **P < 0.01, and ***P < 0.001, ns, not significant. Scale bars, 10 μm.

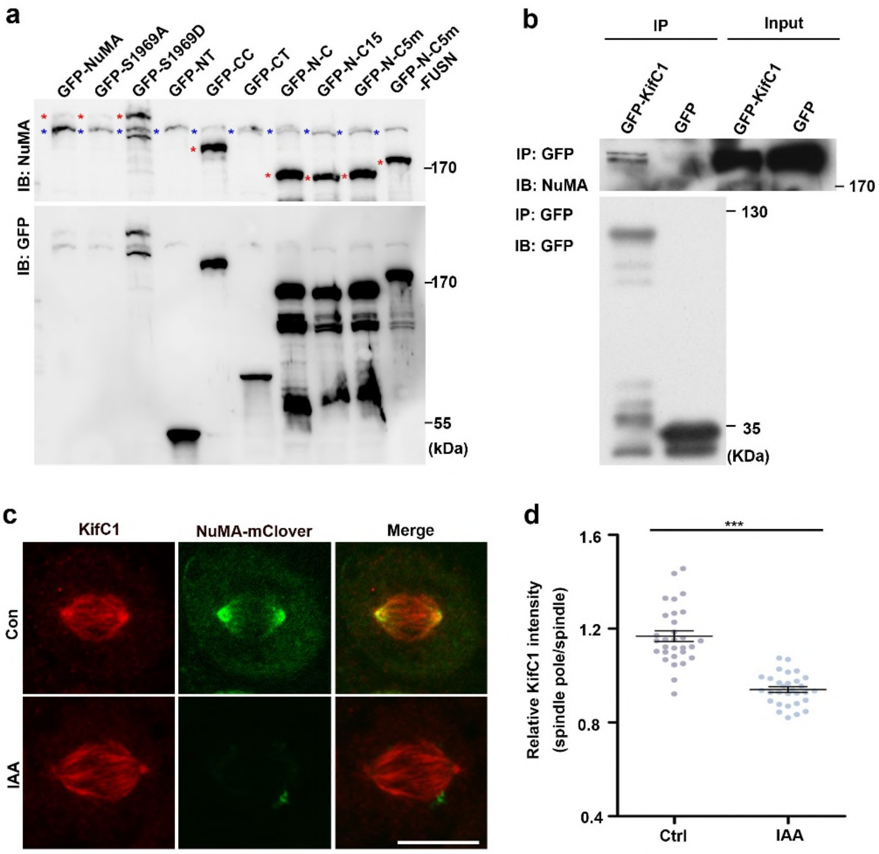

Supplementary Fig. 6 NuMA interacts with KifC1 and affects its localization on spindle poles. a Immunoblotting tested the expressing level of exogenous NuMA truncated mutants. Red asterisks indicated the bands of exogenous NuMA truncated mutants. Blue asterisks indicated the bands of endogenous NuMA. **b** Immunoprecipitation assay showing that KifC1 interacts with NuMA. HEK293T cells were transfected with GFP, GFP-KifC1, arrested in mitosis with nocodazole and processed for IP assay with GFP-Trap beads. **c** Immunofluorescence images of mitotic

spindle in control or IAA-treated NuMA-mACF cells, followed by staining with anti-KifC1 antibody (red). **d** Quantification of the relative KifC1 fluorescence intensity on spindle poles (mean ± SEM, n=30 cells were shown here for one replicated experiment, three independent experiments were repeated). Unpaired two-tailed t test: P<0.0001 for Ctrl/IAA. *P < 0.05, **P < 0.01, and ***P < 0.001, ns, not significant. Scale bars, 10 μm.

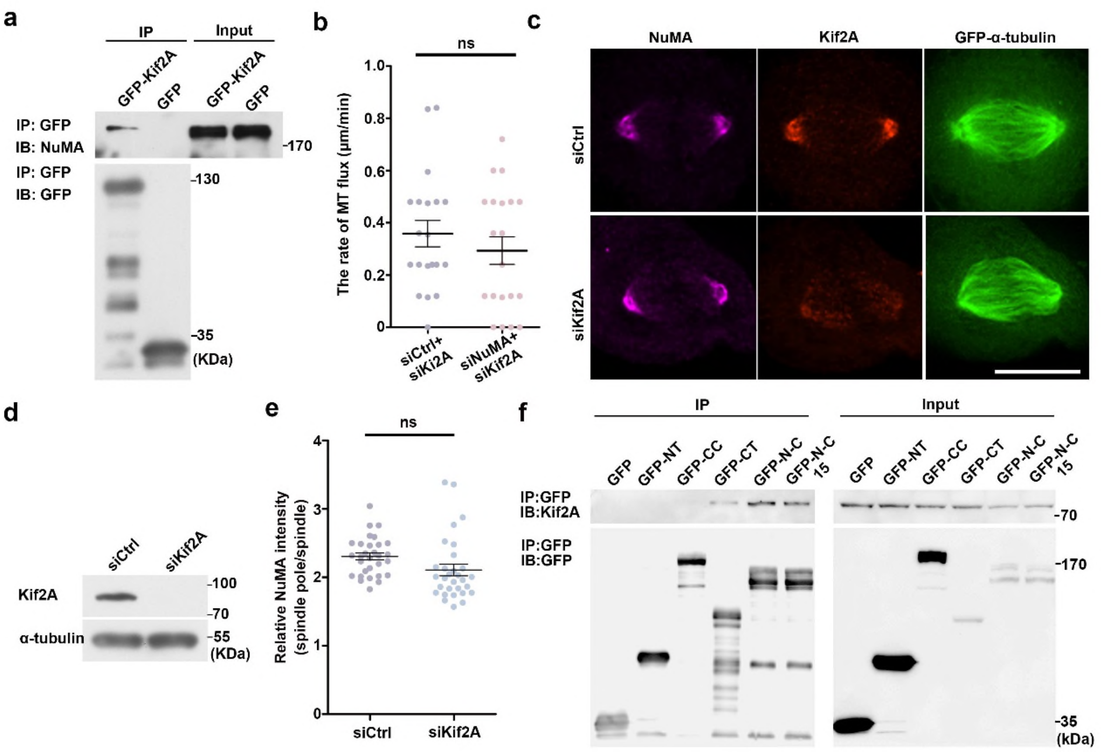

Supplementary Fig. 7 NuMA phase separation regulates spindle dynamics through Kif2A. **a** Immunoprecipitation assay showing that Kif2A interacts with NuMA. HEK293T cells were transfected with GFP, GFP-Kif2A, arrested in mitosis with nocodazole and processed for IP assay with GFP-Trap beads. **b** The mean velocity of poleward spindle microtubule flux in Kif2A-depleted and NuMA/Kif2A-co-depleted cells. The mean velocity was defined by the ratio of the distance that the PAGFP signal travels to the time it takes. Data were presented as mean values ± SEM (n=20 cells were shown here for one replicated experiment, two independent experiments were repeated). Statistical significance, indicated by asterisks, was tested with an unpaired two-tailed t test, P=0.3788 for siCtrl+siKif2A/siNuMA+siKif2A. **c** Immunofluorescence images of

the mitotic spindles in control- and Kif2A-knockdown HeLa cells stably expressing GFP-α-tubulin, followed by staining with anti- Kif2A (red) and NuMA (purple) antibodies. **d** Immunoblotting for Kif2A and α-tubulin expression in control- and Kif2A-siRNA-knockdown HeLa cells. **e** Quantification of the relative NuMA fluorescence intensity on spindle poles in (**c**). Data were presented as mean values ± SEM (n=30 cells were shown here for one replicated experiment, three independent experiments were repeated). Statistical significance, indicated by asterisks, was tested with a two-tailed t test, P=0.0539 for siCtrl/siKif2A. **f** Immunoprecipitation assay showing the interaction between NuMA mutants and Kif2A. HEK293T cells expressing the NuMA mutants were arrested in mitosis with nocodazole and processed for IP assay with GFP-Trap beads. *P < 0.05, **P < 0.01, and ***P < 0.001, ns, not significant. Scale bars, 10 μm.

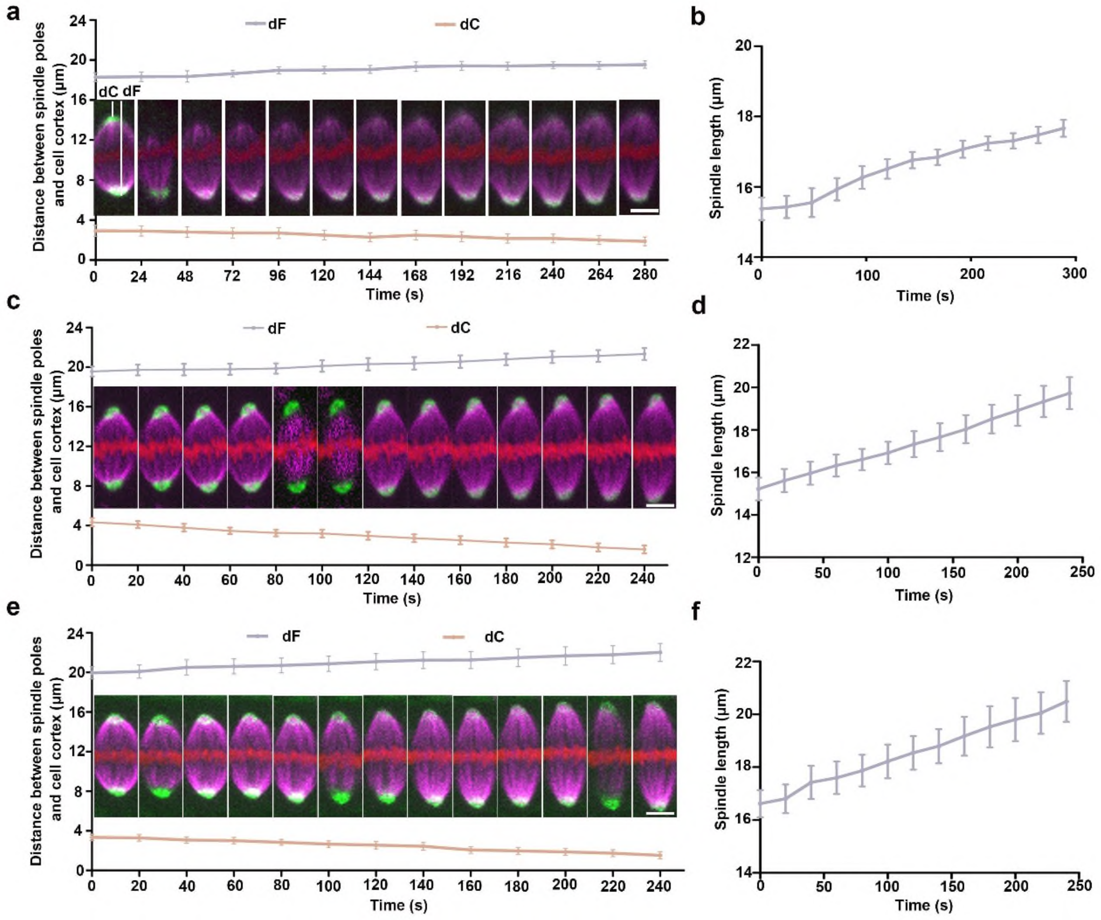

Supplementary Fig. 8 1,6-hexanediol disrupts phase separation of NuMA mutants and elongates spindle length. a, c, e Time-lapse images showing that treatment with 1,6-hexanediol disrupts phase separation of GFP-NuMA (**a**), GFP-S1969A (**c**) or GFP-S1969D (**e**) expressed in HeLa cells and induces elongation of the mitotic spindles. The cells were treated with 5% 1,6-hexanediol, and images were taken every 24 seconds (WT) or 20 seconds (S1969A and S1969D). dC: the distance between the closer pole and the cell cortex. dF: the distance between the farther pole and the cell cortex. Error bars, SEM. n=8 cells. **b, d, f** Quantification showing increases in spindle length in cells treated with 1,6-hexanediol in (**a**), (**c**), (**e**). Error bars, SEM. n=8 cells. Scale bars, 5 μm.
