# Supplementary material for: NuMA regulates mitotic spindle assembly, structural dynamics and function via phase separation
Source: Nat Commun. 2021 Dec 9;12:7157. doi: 10.1038/s41467-021-27528-6 (PMC8660824; doi:10.1038/s41467-021-27528-6)
Supplement: Supplementary file 2 — Description of Additional Supplementary Files [file 41467_2021_27528_MOESM2_ESM.pdf]

Description of Additional Supplementary Files

File name: Supplementary Data 1

Description: Mass spectrometric analyses of NuMAinteracting proteins. Kif2A and KifC1 were identified by MS. PSM: peptide spectrum match, indicating the protein abundance identified in MS.

File name: Supplementary Movie 1

Description: Time-lapse movie of poleward spindle microtubule flux of metaphase spindles in control HeLa cells. HeLa cells stably expressing RFP-H2B (red) were transfected with photoactivatable GFP tagged α-tubulin (PAGFP-αtubulin, green) and control siRNA. Spindle was probed with SiR-tubulin (purple). A 405 nm laser was applied to activate the GFP signal in a rectangular region near the MT plus ends, and the movement of the fluorescent mark (green) was tracked every 10 s. Scale bar, 4.7 µm.

File name: Supplementary Movie 2

Description: Time-lapse movie of poleward spindle microtubule flux of metaphase spindles in NuMA siRNA-treated HeLa cells. HeLa cells stably expressing RFP-H2B (red) were transfected with photoactivatable GFPtagged α-tubulin (PAGFP-α-tubulin, green) and NuMA siRNA. Spindle was probed with SiR-tubulin (purple). A 405 nm laser was applied to activate the GFP signal in a rectangular region near the MT plus ends, and the movement of the fluorescent mark (green) was tracked every 10 s. Scale bar, 5 µm.

File name: Supplementary Movie 3

Description: Time-lapse movie of the formation of endogenous NuMA droplets along with NEBD in normal mitotic NuMA-mACF cells. Endogenous NuMA tagged with mClover (green) forms droplets spontaneously along with NEBD in control NuMA-mACF cells. Images were collected every 40 s. Microtubules were probed with SiR-tubulin (purple). Scale bar, 10 µm.

File name: Supplementary Movie 4

Description: Time-lapse movie of the formation of endogenous NuMA droplets along with NEBD in

nocodazole-treated mitotic NuMAmACF cells. Endogenous NuMA tagged with mClover (green) forms droplets spontaneously along with NEBD in nocodazole-treated NuMAmACF cells. Images were collected every 40 s. Microtubules were probed with SiR-tubulin (purple). Scale bar, 10 µm.

File name: Supplementary Movie 5

Description: Time-lapse movie of NuMA droplet dynamics under treatment with 1,6-hexanediol in normal mitotic NuMA-mACF cells. NuMA-mACF cells, in which endogenous NuMA was tagged by mClover (green), were treated with 10% 1,6-hexanediol, and images were taken every 3 s immediately after the treatment. Scale bar, 10 µm.

File name: Supplementary Movie 6

Description: Time-lapse movie of NuMA droplet dynamics under treatment with 1,6-hexanediol in nocodazole-treated mitotic NuMAmACF cells. NuMA-mACF cells, in which endogenous NuMA was tagged by mClover (green), were treated with nocodazole and 10% 1,6-hexanediol. Images were taken every 3 s immediately after the treatment. Scale bar, 10 µm.

File name: Supplementary Movie 7

Description: Time-lapse movie of the fusion process of endogenous NuMA droplets in nocodazole treated NuMA-mACF cells. Endogenous NuMA (green) droplets fuse randomly in nocodazoletreated NuMA-mACF cells. Images were collected every 40 s. Scale bar, 1 µm.

File name: Supplementary Movie 8

Description: Time-lapse movie of the fission process of endogenous NuMA droplets in nocodazole-treated NuMA-mACF cells. Endogenous NuMA (green) droplets split spontaneously in nocodazole-treated NuMA-mACF cells. Images were collected every 40 s. Scale bar, 1 µm.

File name: Supplementary Movie 9

Description: Time-lapse movie of mitotic progression in HeLa cells transiently expressing GFPNuMA. HeLa cells stably expressing RFP-H2B (red) were transfected with GFP-NuMA (green) and microtubules were probed with SiR-tubulin (purple). Images were taken every 4 min. Scale bar, 10 µm.

File name: Supplementary Movie 10

Description: Time-lapse movie of mitotic progression in HeLa cells transiently expressing GFP-S1969D. HeLa cells stably expressing RFP-H2B (red) were transfected with GFP-S1969D (green) and microtubules were probed with SiR-tubulin (purple). Images were taken every 4 min. Scale bar, 10 µm.

File name: Supplementary Movie 11

Description: Time-lapse movie of mitosis progression in HeLa cells transiently expressing GFPS1969A. HeLa cells stably expressing RFP-H2B (red) were transfected with GFP-S1969A (green) and microtubules were probed with SiR-tubulin (purple). Images were taken every 4 min. Scale bar, 10 µm.

File name: Supplementary Movie 12

Description: Time-lapse movie of dynamic behaviors of Kif2A and NuMA during NEBD in normal NuMA mACF cells. Normal NuMAmACF cells were transfected with RFPKif2A (red) and images were collected every 2 minutes. Microtubules were probed with SiR-tubulin (purple.) Scale bar, 10 µm.

File name: Supplementary Movie 13

Description: Time-lapse movie of dynamic behaviors of Kif2A without endogenous NuMA during NEBD in IAA-terated NuMA-mACF cells. IAA-treated NuMA-mACF cells were transfected with RFP-Kif2A (red) and images were collected every 2 minutes. Microtubules were probed with SiR-tubulin (purple). Scale bar, 10 µm.

File name: Supplementary Movie 14

Description: Time-lapse movie of the elongation of the mitotic spindle treated with 5% 1,6-hexanediol in HeLa cells transiently expressing GFP-NuMA. HeLa cells stably expressing RFP-H2B (red) were transfected with GFP-NuMA (green) and treated with 5% 1,6-hexanediol. Images were taken every 24 s immediately after the treatment. Microtubules were probed with SiRtubulin (purple). Scale bar, 10 µm.

File name: Supplementary Movie 15

Description: Time-lapse movie of the elongation of the mitotic spindle treated with 5% 1,6-hexanediol in HeLa cells transiently expressing GFP-S1969A. HeLa cells stably expressing RFP-H2B (red) were transfected with GFP-1969A (green) and treated with 5% 1,6-hexanediol. Images were taken every 20 s immediately after the treatment. Microtubules were probed with SiR-tubulin (purple). Scale bar, 11 µm.

File name: Supplementary Movie 16

Description: Time-lapse movie of the elongation of the mitotic spindle treated with 5% 1,6-hexanediol in HeLa cells transiently expressing GFP-S1969D. HeLa cells stably expressing RFP-H2B (red) were transfected with GFP-1969D (green) and treated with 5% 1,6-hexanediol. Images were taken every 20 s immediately after the treatment. Microtubules were probed with SiR-tubulin (purple). Scale bar, 10 µm.
