# Supplementary material for: NuMA regulates mitotic spindle assembly, structural dynamics and function via phase separation
Source: Nat Commun. 2021 Dec 9;12:7157. doi: 10.1038/s41467-021-27528-6 (PMC8660824; doi:10.1038/s41467-021-27528-6)
Supplement: Supplementary file 20 — Reporting Summary [file 41467_2021_27528_MOESM20_ESM.pdf]

nature portfolio

Corresponding author(s): Chuanmao Zhang

Last updated by author(s): Nov 9, 2021

Reporting Summary

Nature Portfolio wishes to improve the reproducibility of the work that we publish. This form provides structure for consistency and transparency in reporting. For further information on Nature Portfolio policies, see our Editorial Policies and the Editorial Policy Checklist.

Statistics

For all statistical analyses, confirm that the following items are present in the figure legend, table legend, main text, or Methods section.

n/a	Confirmed	
☐	☒	The exact sample size (*n*) for each experimental group/condition, given as a discrete number and unit of measurement
☐	☒	A statement on whether measurements were taken from distinct samples or whether the same sample was measured repeatedly
☐	☒	The statistical test(s) used AND whether they are one- or two-sided<br>*Only common tests should be described solely by name; describe more complex techniques in the Methods section.*
☒	☐	A description of all covariates tested
☒	☐	A description of any assumptions or corrections, such as tests of normality and adjustment for multiple comparisons
☐	☒	A full description of the statistical parameters including central tendency (e.g. means) or other basic estimates (e.g. regression coefficient) AND variation (e.g. standard deviation) or associated estimates of uncertainty (e.g. confidence intervals)
☐	☒	For null hypothesis testing, the test statistic (e.g. *F*, *t*, *r*) with confidence intervals, effect sizes, degrees of freedom and *P* value noted<br>*Give P values as exact values whenever suitable.*
☒	☐	For Bayesian analysis, information on the choice of priors and Markov chain Monte Carlo settings
☒	☐	For hierarchical and complex designs, identification of the appropriate level for tests and full reporting of outcomes
☒	☐	Estimates of effect sizes (e.g. Cohen's *d*, Pearson's *r*), indicating how they were calculated

Our web collection on statistics for biologists contains articles on many of the points above.

Software and code

Policy information about availability of computer code

Data collection	Confocal imaging was performed on PerkinElmer UltraView VoX spinning disk confocal microscope or Andor Dragonfly spinning disk confocal microscope.<br>Fluorescence images were collected using Applied Precision DeltaVision Elite microscopy.<br>Mass spectrometric detection was performed on Thermo Q Exactive Plus mass spectrometer using a nano-electrospray ion source.<br>The data of size-exclusion chromatography were collected by Unicorn 7 software.
Data analysis	Confocal images acquired on Andor Dragonfly spinning disk confocal microscope were analyzed by Imaris 9.5 software and Volocity 6.1.1 software.<br>Confocal images acquired on PerkinElmer UltraView VoX spinning disk confocal microscope were analyzed by Volocity 6.1.1 software.<br>Mass spectrometric data were alignment with Mouse Reviewed Swiss-Port database by Proteome Discoverer 2.2 software.<br>Statistic analysis was performed on Graphpad Prism 5 software.<br>The data of size-exclusion chromatography were analyzed by Unicorn 7 software.<br>Disorder tendency analysis was performed on PONDR (http://www.pondr.com/)

For manuscripts utilizing custom algorithms or software that are central to the research but not yet described in published literature, software must be made available to editors and reviewers. We strongly encourage code deposition in a community repository (e.g. GitHub). See the Nature Portfolio guidelines for submitting code & software for further information.

Data

Policy information about availability of data

All manuscripts must include a data availability statement. This statement should provide the following information, where applicable:

- Accession codes, unique identifiers, or web links for publicly available datasets
- A description of any restrictions on data availability
- For clinical datasets or third party data, please ensure that the statement adheres to our policy

All relevant data supporting the key findings of this study are available within the article, supplementary information and source data. Source data are provided with this paper. Biological materials including cell lines and custom antibodies generated for this study will be shared upon request within the limits of respective material transfer agreements for as long as they are available in the laboratory.

Field-specific reporting

Please select the one below that is the best fit for your research. If you are not sure, read the appropriate sections before making your selection.

☒ Life sciences ☐ Behavioural & social sciences ☐ Ecological, evolutionary & environmental sciences

For a reference copy of the document with all sections, see nature.com/documents/nr-reporting-summary-flat.pdf

Life sciences study design

All studies must disclose on these points even when the disclosure is negative.

Sample size	The sample size was determined based on the minimum requirement to perform statistical tests and the availability of materials.
Data exclusions	No data or subjects were excluded from the analysis.
Replication	All findings were confirmed by at least two independent experiments.
Randomization	No strategy for randomization and/or stratification was employed, as cell culture experiments are not subjected to randomization.
Blinding	No blind experiments was used in this study because the same investigator designed and conducted the experiments by setting proper controls.

Reporting for specific materials, systems and methods

We require information from authors about some types of materials, experimental systems and methods used in many studies. Here, indicate whether each material, system or method listed is relevant to your study. If you are not sure if a list item applies to your research, read the appropriate section before selecting a response.

Materials & experimental systems

n/a	Involved in the study	
☐	☒	Antibodies
☐	☒	Eukaryotic cell lines
☒	☐	Palaeontology and archaeology
☒	☐	Animals and other organisms
☒	☐	Human research participants
☒	☐	Clinical data
☒	☐	Dual use research of concern

Methods

n/a	Involved in the study	
☒	☐	ChIP-seq
☒	☐	Flow cytometry
☒	☐	MRI-based neuroimaging

Antibodies

Antibodies used

Antibodies used were listed as antibody name, source and identifier.
Mouse monoclonal antibody to α-tubulin, Sigma-Aldrich, Cat# T9026, Lot# 078M4796V, Clone DM1A ascites fluid, RRID: AB_477593, 1:500 for IF, 1:1500 for WB
Rabbit polyclonal antibody to α-tubulin, Abcam, Cat# ab18251, Lot# GR3199234-1, AB_2210057, 1:200 for IF
Rabbit polyclonal antibody to NuMA, Abcam Cat# ab97585, RRID:AB_10680001, 1: 250 for IF, 1:1000 for WB
Mouse monoclonal antibody to NuMA, Santa Cruz Biotechnology Cat# sc-365532, RRID:AB_10846197, 1:1000 for WB
Mouse monoclonal antibody to Kif2A, Santa Cruz Biotechnology Cat# sc-271471, Lot# H0415, RRID:AB_10655537, 1:200 for IF, 1: 1000 for WB
Mouse polyclonal antibody to KifC1, in-house generation, 1: 100 for IF, 1: 500 for WB

Mouse monoclonal antibody to pT288-Aurora A, Cell Signaling Technology Cat# 3079, Lot# 5, RRID:AB_2061481, 1:200 for IF
Mouse monoclonal antibody to GFP, Abmart Cat# M20004, Lot# 214574, RRID:AB_2619674, 1:5000 for WB
Rabbit monoclonal antibody to p-Aurora A/B/C, CST Cat#2914S, Lot# 9, RRID:AB_2061631, 1:1000 for WB
Rabbit polyclonal antibody to γ-tubulin, Sigma-Aldrich Cat# T3559, RRID:AB_477575, 1:500 for IF
Mouse monoclonal antibody to GAPDH, Proteintech Cat# 60004-1-Ig, Lot# 10017731, RRID:AB_2107436, 1:5000 for WB
Mouse IgG (H+L) antibody, Alexa Fluor 488 Conjugated, Invitrogen, Cat# A-21202, Lot# 2229195, RRID:AB_141607, 1:200 for IF
Rabbit IgG (H+L) antibody, Alexa Fluor 488 Conjugated, Invitrogen, Cat# A-21206, Lot# 2156521, RRID:AB_2535792, 1:200 for IF
Mouse IgG (H+L) antibody, Alexa Fluor 594 Conjugated, Invitrogen, Cat# A-21203, Lot# 2294985, RRID:AB_141633, 1:200 for IF
Rabbit IgG (H+L) antibody, Alexa Fluor 594 Conjugated, Invitrogen, Cat# A-21207, Lot# 2221800, RRID:AB_141637, 1:200 for IF
Mouse IgG (H+L) antibody, Alexa Fluor 647 Conjugated, Invitrogen, Cat# A32728, Lot# TE266004, RRID:AB_2633277, 1:200 for IF
Rabbit IgG (H+L) antibody, Alexa Fluor 647 Conjugated, Invitrogen,Cat# A-31573, Lot# 2083195, RRID:AB_2536183, 1:200 for IF

Validation

Mouse monoclonal antibody to α-tubulin, Sigma-Aldrich,Cat# T9026: bovine, rat, yeast, human, mouse, chicken, fungi, amphibian; IF, WB. (https://www.sigmaaldrich.cn/CN/zh/product/sigma/t9026?context=product)
Rabbit polyclonal antibody to α-tubulin, Abcam, Cat# ab18251: mouse, rat, human; ICC/IF, Flow Cyt, WB. (https://www.abcam.cn/alpha-tubulin-antibody-microtubule-marker-ab18251.html)
Rabbit polyclonal antibody to NuMA, Abcam Cat# ab97585: human; WB, IHC-P, ICC/IF. (https://www.abcam.cn/numa-antibody-ab97585.html)
Mouse monoclonal antibody to NuMA, Santa Cruz Biotechnology Cat# sc-365532: human; WB, IP, IF, IHC(P) and ELISA. (https://www.scbt.com/p/numa-antibody-f-11?requestFrom=search)
Mouse monoclonal antibody to Kif2A, Santa Cruz Biotechnology Cat# sc-271471: mouse, rat and human; WB, IP, IF and ELISA. (https://www.scbt.com/p/kif2a-antibody-d-7?requestFrom=search)
Mouse monoclonal antibody to pT288-Aurora A, Cell Signaling Technology Cat# 3079: human; WB, IF. (https://www.cellsignal.cn/products/primary-antibodies/phospho-aurora-a-thr288-c39d8-rabbit-mab/3079?site-search-type=Products&N=4294956287&Ntt=3079&fromPage=plp&_requestid=1965823)
Mouse monoclonal antibody to GFP, Abmart Cat# M20004: All; WB,IF,IHC,IP. (http://www.ab-mart.com.cn/page.aspx?node=%2060%20&id=%20971)
Rabbit monoclonal antibody to p-Aurora A/B/C, CST Cat#2914S: human, mouse and rat; WB, IF, FACS. (https://www.cellsignal.cn/products/primary-antibodies/phospho-aurora-a-thr288-aurora-b-thr232-aurora-c-thr198-d13a11-xp-rabbit-mab/2914?site-search-type=Products&N=4294956287&Ntt=2914s&fromPage=plp&_requestid=1967784)
Rabbit polyclonal antibody to γ-tubulin, Sigma-Aldrich Cat# T3559: chicken, human; IF, WB, ARR. (https://www.sigmaaldrich.cn/CN/zh/product/sigma/t3559?context=product)
Mouse monoclonal antibody to GAPDH, Proteintech Cat# 60004-1-Ig: human, mouse, rat, yeast, plant, zebrafish; WB, IP, IHC, IF, FC, CoIP, ChIP. (https://www.ptglab.com/products/GAPDH-Antibody-60004-1-Ig.htm#product-information)
Homemade mouse polyclonal anti-KifC1 was validated by SDS-PAGE and by its decreasing signal (full length KifC1) when endogenous KifC1 was interfered by specific siRNA, data were shown in Figure 5i.

Eukaryotic cell lines

Policy information about cell lines

Cell line source(s)

Cell lines used were listed as name, source and identifier.
HEK293T, ATCC, CRL-11268
HeLa, ATCC, CCL-2
NuMA-mACF cell line (HCT-116), gift from Dr. Tomomi Kiyomitsu (Nagoya University, Japan), N/A
HeLa-GFP-NuMA, this paper, N/A
NuMA-mACF-mKate-NuMA, this paper, N/A
NuMA-mACF-mKate-NC, this paper, N/A
NuMA-mACF-mKate-NC15, this paper, N/A
NuMA-mACF-mKate-S1969A, this paper, N/A
NuMA-mACF-mKate-S1969D, this paper, N/A

Authentication

HeLa and HEK293T have been validated by the suppliers. The growth of the cell lines were verified with the supplier's data sheets with stable morphology feature and pharmacological response. NuMA-mACF cell line (HCT-116) was gifted from Dr. Tomomi Kiyomitsu. This cell line was not authenticated by us.

Mycoplasma contamination

No any mycoplasma contamination.

Commonly misidentified lines
(See ICLAC register)

There were no misidentified lines.
